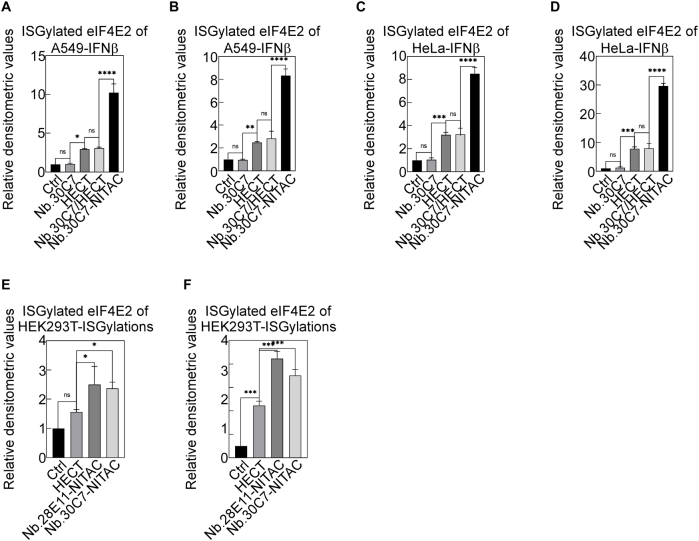# NITAC-mediated ISGylation of eIF4E2 attenuates GSK3β proline-directed kinase activity, conferring cytoprotection

**DOI:** 10.1016/j.jbc.2025.110777

**Published:** 2025-09-27

**Authors:** Lan Li, Jinjin Gong, Huiting Liang, Ying Yang, Yuanshun Wu, Ziyi Yin, Anni Wang, Shaoxiang Luo, Jian Chen, Min Zhang

**Affiliations:** 1 College of Biomedicine and Health, College of Life Science and Technology, Huazhong Agricultural University, Wuhan, China; 2 Wuhan Huamei Biotech Co., Ltd, Wuhan, China; 3 Department of Head and Neck Surgery, Hubei Cancer Hospital, Tongji Medical College, Wuhan, China

**Keywords:** NITAC, ISGylation, eIF4E2-GSK3β, proline-directed kinase, OGD/R, cytoprotection

## Abstract

Eukaryotic translation initiation factor 4E family member 2 (eIF4E2) has recently been identified as an interacting protein of GSK3β and regulates its proline-directed kinase activity. eIF4E2 undergoes ISGylation at K134 and K222, a conserved posttranslational modification mediated by interferon-stimulated gene 15. In this study, we engineered a novel Nanobody-based ISGylation Targeting Chimera (NITAC) tool to specifically activate eIF4E2 ISGylation and investigate its role in the eIF4E2–GSK3β signaling pathway. By integrating eIF4E2-specific nanobodies Nb.30C7 with the catalytic E3 ligase domain HECT from HERC5, we constructed the NITAC (Nb.30C7-HECT). This NITAC tool mediates site-specific ISGylation of eIF4E2, enhancing the eIF4E2–GSK3β interaction and unexpectedly suppressing proline-directed serine/threonine phosphorylation across multiple crucial targets within the eIF4E2–GSK3β pathway. Importantly, NITAC treatment exerted cytoprotection against oxygen-glucose deprivation/reoxygenation stress, a commonly used *in vitro* model to simulate ischemic conditions in cell cultures. Furthermore, NITAC treatment reduced reactive oxygen species in neurons and microglia and promoted an anti-inflammatory phenotype in microglia by suppressing proline-directed serine/threonine phosphorylation. In summary, we created a novel NITAC to specifically activate eIF4E2 ISGylation, which showed cytoprotective effects under oxygen-glucose deprivation/reoxygenation stress by inhibiting GSK3β proline-directed kinase activity.

Glycogen synthase kinase 3β (GSK3β) is a highly conserved serine/threonine kinase involved in various diseases, including ischemic stroke, neurodegenerative disorders, and cancer (1). As a key regulator, GSK3β influences glycogen metabolism, cell cycle progression, and embryonic development by phosphorylating numerous protein substrates (2). GSK3β exhibits two phosphorylation modes: proline-directed phosphorylation (S/T-P or S/T-X-P, where X is any amino acid) and preference for prephosphorylated proteins (S/T-X-X-X-S/T(P), where the second S/T is phosphorylated) (3). Despite extensive research, understanding the differential substrate phosphorylation mechanisms of GSK3β remains limited. Eukaryotic translation initiation factor 4E2 (eIF4E2) is a homolog of eIF4E and serves as a crucial mediator in the initiation of translation in response to hypoxic stress (4). Previous research has shown that eIF4E2 directly interacts with GSK3β, thereby activating GSK3β′s S/T-P kinase activity (5). This activation enhances the phosphorylation of RBM38-Ser195, Hif1α-Ser589, and p53-Ser315, all of which are proline-directed residues (5). The eIF4E2–GSK3β pathway protects cells from hypoxia-induced liver damage by inhibiting cellular senescence under physiological hypoxia conditions (5).

ISGylation is a posttranslational modification process involving the covalent attachment of the ubiquitin-like protein ISG15 to target proteins (6). This process coordinates immune responses and cell homeostasis regulation under various physiological and pathological conditions, particularly during viral infections, type I interferon responses, and inflammatory challenges (7). ISGylation is associated with the regulation of several neurological diseases, including stroke, traumatic brain injury, basal ganglia calcification, and ataxia-telangiectasia syndrome (8, 9). Recent studies have demonstrated that transient ischemic attacks significantly increase ISGylation of proteins in the cortical regions of mouse brains (6, 10, 11). Transgenic mice deficient in ISGylation exhibit exacerbated ischemic brain injury, suggesting that ISGylation might serve as an endogenous neuroprotective response following ischemic insults (6). However, the specific molecular mechanisms by which ISGylation regulates ischemic stroke pathology remain unclear.

GSK3β is considered a critical therapeutic target for ischemic stroke, as inhibiting it can protect the nervous system from oxidative stress or inflammation-mediated damage (1, 12, 13, 14). However, the underlying mechanisms are not fully understood. Interestingly, eIF4E2 is subject to ISGylation at lysine residues K134 and K222, with K222 being close to the GSK3β-binding domain (231–242aa) of eIF4E2 (5, 15). Thus, eIF4E2's ISGylation could potentially modulate GSK3β kinase activity and may regulate the eIF4E2-GSK3β pathway, playing a critical role in ischemic stroke. Developing specific tools to activate eIF4E2 ISGylation is crucial. Current methods for activating ISGylation usually involve interferon stimulation or cotransfection of the ISG15 system, which includes the E1 activating enzyme, E2 conjugating enzyme, E3 ligase, and ISG15 (16). These approaches often face limitations due to interferon sensitivity and inefficiencies in multiplasmid transfection, as well as insufficient specificity, leading to broad and multitargeted substrate ISGylation. Specific substrate-targeted ISGylation activation has not yet been reported.

The current technological limitations hinder our understanding of specific protein ISGylation in different cellular environments and disease states, highlighting the urgent need for more precise molecular tools. Recent advances in targeted protein modification strategies, particularly the development of proteolysis-targeting chimera (PROTACs/bioPROTACs), offer new solutions to this challenge (17, 18). For example, bioPROTACs utilize intrabodies such as nanobodies to replace the native substrate recognition domain of E3 ligases, enabling specific protein ubiquitination and degradation (18).

Here, we propose a new tool, Nanobody-based ISGylation Targeting Chimera (NITAC), to specifically activate ISGylation of target eIF4E2. Specifically, we leverage the high specificity and affinity of nanobodies to integrate eIF4E2-specific nanobodies Nb.30C7 into the catalytic E3 ligase domain HECT from HERC5. This NITAC can selectively induce ISGylation of the target protein eIF4E2. Interestingly, NITAC-mediated ISGylation of eIF4E2 inhibits the S/T-P kinase activity of GSK3β, providing significant cytoprotection under oxygen-glucose deprivation/reoxygenation (OGD/R) conditions.

## Result

### Identification of nanobodies specific to eIF4E2

Recently, the bioPROTAC method for achieving targeted protein degradation has been developed, demonstrating that intracellular nanobodies can direct E3 ligases to specific protein modifications (18). To investigate the role of ISGylation of eIF4E2, we plan to develop tools that mediate specific ISGylation by fusing an eIF4E2-targeting nanobody with an E3 ligase known to mediate ISGylation. Specifically, we strategically chose peptide epitopes corresponding to evolutionarily conserved regions at the N and C termini of eIF4E2 for nanobody development. The N-terminal peptide (e2-R: MNNKFDALKDDDSGD) and C-terminal peptide (e2-I: RLLFQNLWKPRL) were selected based on their spatial relationship to the ISGylation site, with e2-R positioned distally and e2-I proximally. These peptides were conjugated to bovine serum albumin (BSA) to generate BSA-peptide conjugates for nanobody screening. We engineered a synthetic yeast-display nanobody library with a theoretical diversity of 2.5 × 10^9^ unique sequences. The BSA-peptide conjugates were immobilized on carboxylated magnetic beads for subsequent selection procedures.

**Figure 1:**
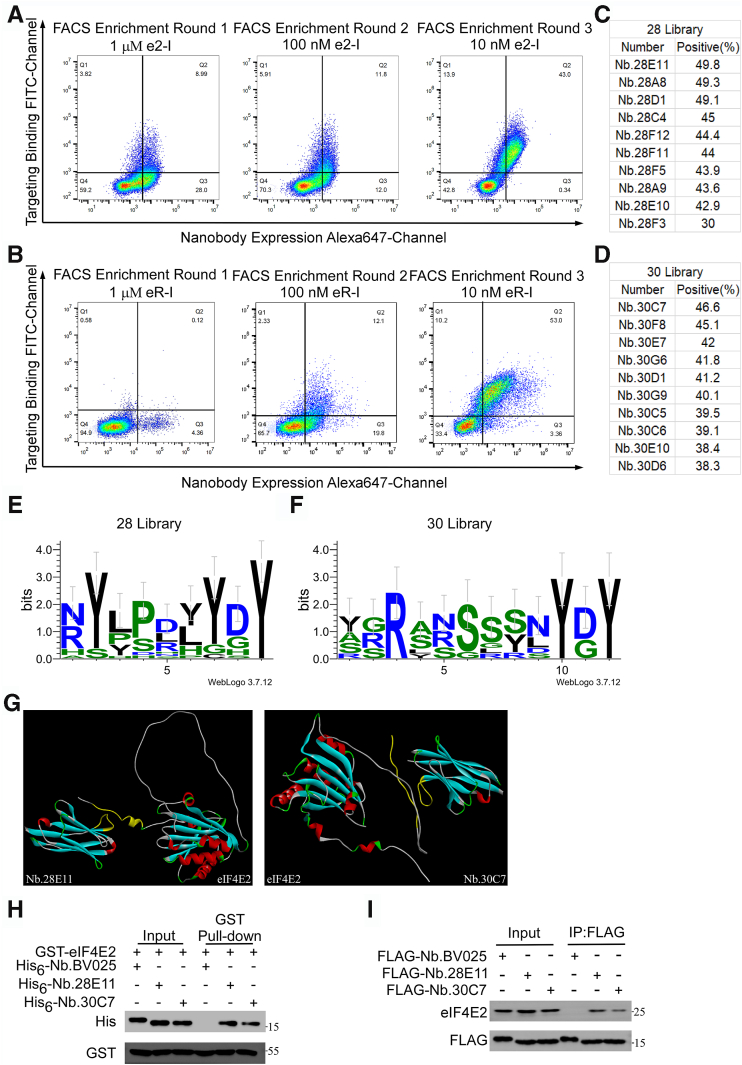
**Identification of nanobodies specific to eIF4E2.***A* and *B*, identification of nanobodies recognizing the C-terminal (*A*) and N-terminal (*B*) regions of eIF4E2 after three rounds of FACS. Antigen concentrations were decreased sequentially over three rounds of FACS, from 1 μM, 100 nM, to 10 nM. *C* and *D*, flow cytometry analysis of the top 10 nanobodies ranked by antigen binding affinity. Monoclonal yeast from the final round of FACS were cultured in 96-deep-well plates, expressed, and incubated with the antigen (1 nM), followed by flow cytometry to determine binding rates. *E* and *F*, sequence similarity analysis of enriched nanobody CDR3 regions. The final FACS products were analyzed by next-generation sequencing, and sequence alignments of the most enriched nanobody CDR3 regions were performed using the WebLogo online tool. *G*, molecular docking of nanobodies with eIF4E2. The two nanobodies with the highest antigen binding rates, Nb.28E11 and Nb.30C7, which demonstrated significant enrichment in next-generation sequencing, were subjected to molecular docking with eIF4E2 using the HADDOCK online tool. The interaction regions are highlighted in *yellow*. *H*, GST pull-down assay showing direct binding of nanobodies Nb.28E11 and Nb.30C7 to eIF4E2. Beads bound with GST-eIF4E2 were incubated with purified His_6_-tagged nanobodies, followed by IB analysis. *I*, coimmunoprecipitation (Co-IP) results demonstrating the interaction of nanobodies Nb.28E11 and Nb.30C7 with eIF4E2 in cells. HEK293T cells were transfected with plasmids expressing FLAG-Nb.28E11 and FLAG-Nb.30C7 for 48 h. Cell lysates were incubated with FLAG beads, followed by IB analysis. FACS, fluorescence-activated cell sorting; eIF4E2, eukaryotic translation initiation factor 4E2; GST, glutathione-*S*-transferase; IB, immunoblot.

The isolation of high-affinity nanobodies targeting the conserved termini of eIF4E2 was achieved through a systematic enrichment strategy combining two rounds of magnetic-activated cell sorting (MACS) followed by three rounds of fluorescence-activated cell sorting (FACS). Flow cytometric analysis identified dual-fluorescent positive populations (Alexa 647+/FITC+) in quadrant Q2, which were subsequently cultured on SD/-Trp selective media. Each enrichment cycle utilized the previously enriched library as input for subsequent FACS iterations, enabling progressive selection of high-affinity clones. The proportion of dual-positive populations increased substantially from initial frequencies of 8.99% and 0.12% to 43% and 53%, respectively (Fig. 1, *A* and *B*), demonstrating robust enrichment efficiency. Among the isolated clones, Nb.28E11 and Nb.30C7 exhibited superior binding affinities toward the C- and N-terminal regions of eIF4E2, respectively (Fig. 1, *C* and *D*).

Next-generation sequencing analysis revealed that the top 30 enriched nanobodies, including Nb.28E11 and Nb.30C7, constituted over 80% of the successfully assembled nanobody repertoire. Sequence analysis of 10 selected nanobodies with conserved CDR3 lengths was performed using WebLogo 3.7.4 to evaluate CDR3 sequence conservation patterns (Fig. 1, *E* and *F*). *In silico* molecular docking simulations using HADDOCK predicted the binding interfaces between Nb.28E11/Nb.30C7 and eIF4E2, with interaction regions highlighted in yellow (Fig. 1*G*). The predicted interactions were experimentally validated through glutathione-*S*-transferase (GST) pull-down assays, demonstrating specific binding between GST-eIF4E2 and His_6_-tagged Nb.28E11/Nb.30C7, while the control nanobody His_6_-Nb.BV025 showed no interaction (Fig. 1*H*). Nb.BV025 was selected as a negative control based on its favorable expression characteristics and documented lack of antigen-binding activity (Yu *et al.*, 2020).

To validate these interactions in a physiologically relevant context, we performed coimmunoprecipitation assays in HEK293T cells expressing FLAG-tagged nanobodies. Consistent with *in vitro* findings, FLAG-Nb.28E11 and FLAG-Nb.30C7 specifically coprecipitated with endogenous eIF4E2, while FLAG-Nb.BV025 showed no detectable interaction (Fig. 1*I*).

### Identification of an E3 ligase mediating ISGylation of eIF4E2

**Figure 2:**
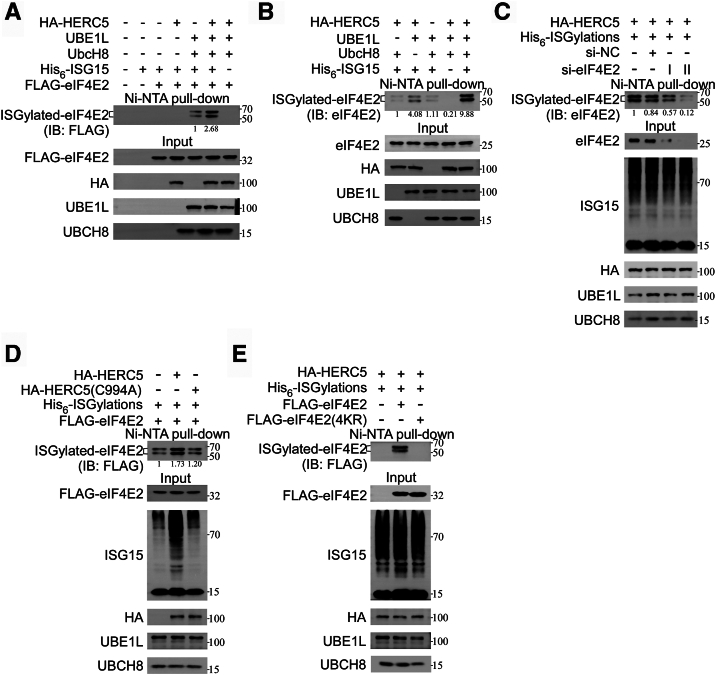
**Identification of an E3 ligase mediating ISGylation of eIF4E2.***A* and *B*, ISGylation of eIF4E2. *A*, HEK293T cells were transfected with plasmids expressing HA-HERC5, FLAG-eIF4E2, UBE1L, UBCH8, and His_6_-ISG15 as indicated. After 48 h, cell lysates were subjected to Ni-NTA pull-down and IB analysis with anti-FLAG antibodies. The positions of unmodified and ISGylated eIF4E2 are shown on the *right*. *B*, HEK293T cells were transfected with plasmids expressing HA-HERC5, UBE1L, UBCH8, and His_6_-ISG15 as indicated. Cell lysates were subjected to Ni-NTA pull-down and IB analysis. *C*, HEK293T cells were transfected with siRNA-targeting *eIF4E2* (si-*eIF4E2*) or a control siRNA (si-NC), along with plasmids expressing HA-HERC5 and the His_6_-ISGylation system (UBE1L, UBCH8, and His_6_-ISG15). Cell lysates were subjected to Ni-NTA pull-down and IB analysis. *D*, either HA-HERC5 (WT or C994A mutant) was transfected to HEK293T cells with the His6-ISGylation system and FLAG-eIF4E2, followed by Ni-NTA pull-down and IB analysis. *E*, either Flag-eIF4E2 (WT or mutant) was transfected to HEK293T cells with the His6-ISGylation system and HA-HERC5, followed by Ni-NTA pull-down and IB analysis. eIF4E2, eukaryotic translation initiation factor 4E2; IB, immunoblot.

To develop effective NITAC, a first step involves selecting a more efficient E3 ligase for mediating the ISGylation of eIF4E2. The RBR E3 ubiquitin ligase HHARI is known to facilitates the ISGylation of eIF4E2. However, considering that HHARI has been primarily implicated in ubiquitination, other E3 enzymes should be considered. Among them, HERC5, which is the predominant ISG15 E3 ligase in human cells due to its clear specificity for ISG15 binding and well-characterized catalytic mechanism, may also mediate the ISGylation of eIF4E2. Target protein ISGylation can typically be achieved by cotransfecting noninterferon-responsive cells (*e.g.*, HEK293T) with four plasmids expressing the core ISG15 conjugation machinery components: *ISG15*, *UBE1L* (E1), *UBE2L6* (E2, formerly *UbcH8*), and *HERC5* (E3 ligase). We found that overexpression of His_6_-ISGylation system, involving E1, E2, His_6_-tagged ISG15, resulted in the appearance of two shifted bands with molecular weights around 50 to 70 kDa in Flag-tagged eIF4E2, indicating ISGylation (Fig. 2*A*). In this system, the inclusion of HERC5 further increased the ISGylation (Fig. 2*A*). The absence of His_6_-tagged ISG15 in the His_6_-ISGylation system prevented the appearance of these bands, even in the presence of HERC5 (Fig. 2*A*). Similarly, overexpression of His_6_-ISGylation system and HERC5 promoted the ISGylation of endogenous eIF4E2, as evidenced by the presence of two shifted bands (Fig. 2*B*). As expected, knockdown of *eIF4E2* using two different siRNAs led to a decrease in eIF4E2 expression, along with a reduction in the intensity of these two shifted bands, due to the presence of His_6_-ISGylation system and HERC5 (Fig. 2*C*).

Furthermore, we further determined the role of HERC5 in mediating eIF4E2 ISGylation. We found that the inclusion of HERC5 into His_6_-ISGylation system significantly increased the ISGylation of eIF4E2, whereas the HERC5 mutant carrying the C994A substitution, which impairs its ligase activity, failed to enhance the ISGylation of eIF4E2 (Fig. 2*D*). Additionally, the expression His_6_-ISGylation system and HERC5 had no impact on the ISGylation of the eIF4E2(4KR) mutant, which lacks potential ISGylation sites (K121R, K130R, K134R, K222R) (Fig. 2*E*). Collectively, these findings demonstrate that eIF4E2 undergoes ISGylation, and HERC5 is one of the key E3 ligase candidates for mediating the ISGylation of eIF4E2.

### NITAC-enabled activation of eIF4E2 ISGylation

**Figure 3:**
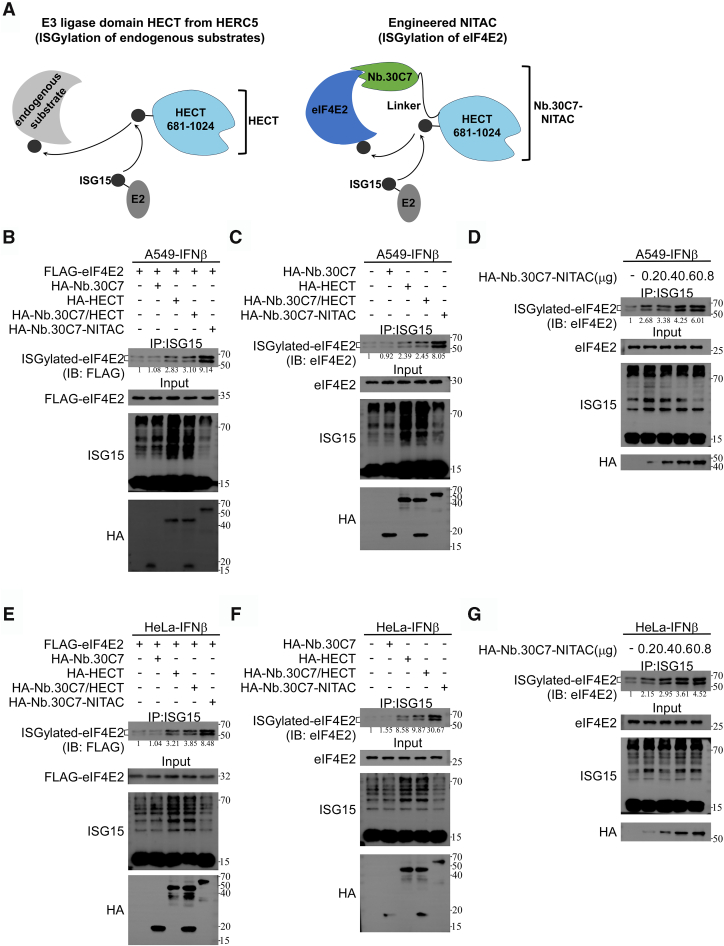
**NITAC-enabled activation of eIF4E2 ISGylation.***A*, schematic diagram of the Nb.30C7-NITAC structure. *B* and *E*, Nb.30C7-NITAC enhanced the ISGylation of Flag-eIF4E2. *B*, A549 or (*E*) HeLa cells were transfected with plasmids expressing FLAG-eIF4E2, HA-Nb.30C7, HA-HECT, HA-Nb.30C7/HA-HECT and HA-Nb.30C7-NITAC as indicated for 12 h and then treated with IFNβ (1000 U/ml) for another 36 h. Then, cell extracts were subjected to Co-IP with anti-ISG15 antibody, followed by IB analysis. *C* and *F*, Nb.30C7-NITAC enhances the ISGylation of endogenous eIF4E2. *C*, A549 or (*F*) HeLa cells were transfected with plasmids expressing HA-Nb.30C7, HA-HECT, HA-Nb.30C7/HA-HECT, and HA-Nb.30C7-NITAC as indicated for 12 h and then treated with IFNβ (1000 U/ml) for another 36 h. Then, cell extracts were subjected to Co-IP with anti-ISG15 antibody, followed by IB analysis. *D* and *G*, Nb.30C7-NITAC enhances the ISGylation of exogenous eIF4E2 in a dose-dependent manner. *D*, A549 or (*G*) HeLa cells were transfected with plasmids (0, 0.2, 0.4, 0.6, or 0.8 μg) expressing HA-Nb.30C7-NITAC for 12 h and then treated with IFNβ (1000 U/ml) for another 36 h. Then, cell extracts were subjected to Co-IP with anti-ISG15 antibody, followed by IB analysis. Co-IP, coimmunoprecipitation; eIF4E2, eukaryotic translation initiation factor 4E2; IB, immunoblot; IFNβ, interferon β; NITAC,Nanobody-based ISGylation Targeting Chimera.

**Supporting Figure S1:**
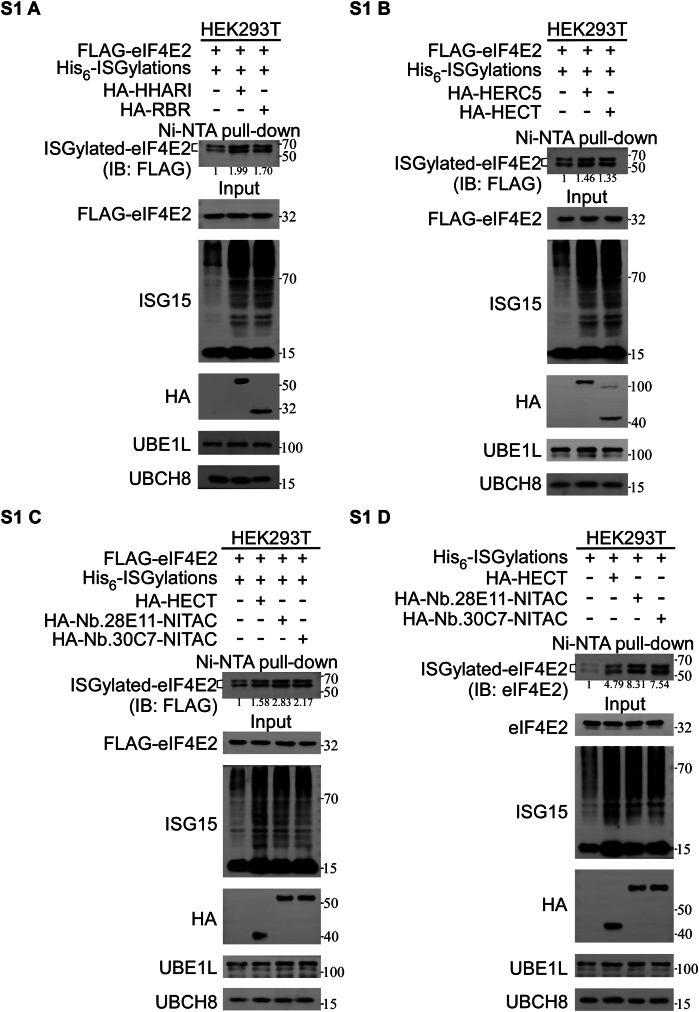


Leveraging the specificity of nanobodies, we aimed to guide the HECT domain derived from HERC5 to precisely regulate eIF4E2 ISGylation. In HEK293T cells overexpressing the His_6_-ISGylation system (UBE1L, UBCH8, and His_6_-ISG15), we simultaneously overexpressed the HECT domain of HERC5 or the full-length HERC5 protein, as well as the RBR domain of HHARI or the full-length HHARI protein. We observed that the HECT/RBR domains of HERC5/HHARI E3 ligases directly promoted eIF4E2 ISGylation, albeit with lower efficacy than the full-length HERC5/HHARI E3 ligase (Fig. S1, *A* and *B*). Subsequently, we constructed NITAC tools by fusing the HECT domain to nanobodies Nb.30C7 and Nb.28E11 *via* a flexible linker (SSSGS) (Fig. 3*A*). In HEK293T cells overexpressing the His_6_-ISGylation system, we coexpressed the HECT domain or Nb.30C7/Nb.28E11-NITAC constructs. The results revealed that NITAC enhanced the ISGylation of Flag-tagged eIF4E2 in the presence of the ISGylation system, demonstrating improved efficiency compared to HECT domain overexpression alone (Fig. S1*C*). Notably, NITAC successfully activated the ISGylation of endogenous eIF4E2 (Fig. S1*D*). We observed that the HECT domain alone could also induce endogenous eIF4E2 ISGylation, potentially due to the high baseline ISGylation levels in HEK293T cells overexpressing the His_6_-ISGylation system. However, these results demonstrate that using nanobodies to direct the HECT domain toward substrate-specific ISGylation remains a feasible strategy.

To further validate the effectiveness and specificity of the NITAC approach, we selected A549 and HeLa cells due to their relatively low basal ISGylation levels (19, 20, 21). In addition, interferon β (IFNβ) treatment was employed to activate protein ISGylation in interferon-sensitive cell lines, such as HeLa and A549 (22). Given the spatial proximity of Nb.28E11 binding to the critical K222 ISGylation site (residues 231–242) that might interfere with posttranslational modification dynamics, we prioritized Nb.30C7 for subsequent studies. This nanobody specifically recognizes the N-terminal domain (residues 1–15) of eIF4E2, distal from known functional domains. By engineering a fusion construct linking Nb.30C7 to the catalytic HECT domain, we generated the Nb.30C7-NITAC system for targeted ISGylation modulation. The expression of the HECT domain alone or the coexpression of Nb.30C7 and HECT (Nb.30C7/HECT) significantly increased global ISGylation levels and moderately activated eIF4E2 ISGylation (Fig. 3, *B* and *C*). In contrast, Nb.30C7-based NITAC (Nb.30C7-NITAC) specifically and robustly activated eIF4E2 ISGylation, while showing minimal effects on global ISGylation levels (Fig. 3, *B* and *C*). Notably, Nb.30C7 alone did not significantly affect eIF4E2 ISGylation levels. Furthermore, Nb.30C7-NITAC induced eIF4E2 ISGylation in a dose-dependent manner in IFNβ-treated A549 cells, with no significant changes in overall ISGylation levels (Fig. 3*D*). Similarly, Nb.30C7-NITAC showed robust activation of eIF4E2 ISGylation in IFNβ-treated HeLa cells (Fig. 3, *E*–*G*). Collectively, we have established and validated a nanobody-E3 chimera tool that exhibits specific and potent activation of eIF4E2 ISGylation, providing a novel approach to investigate the role of eIF4E2 ISGylation.

### ISGylation of eIF4E2 enhances its interaction with GSK3β

**Supporting Figure S2:**
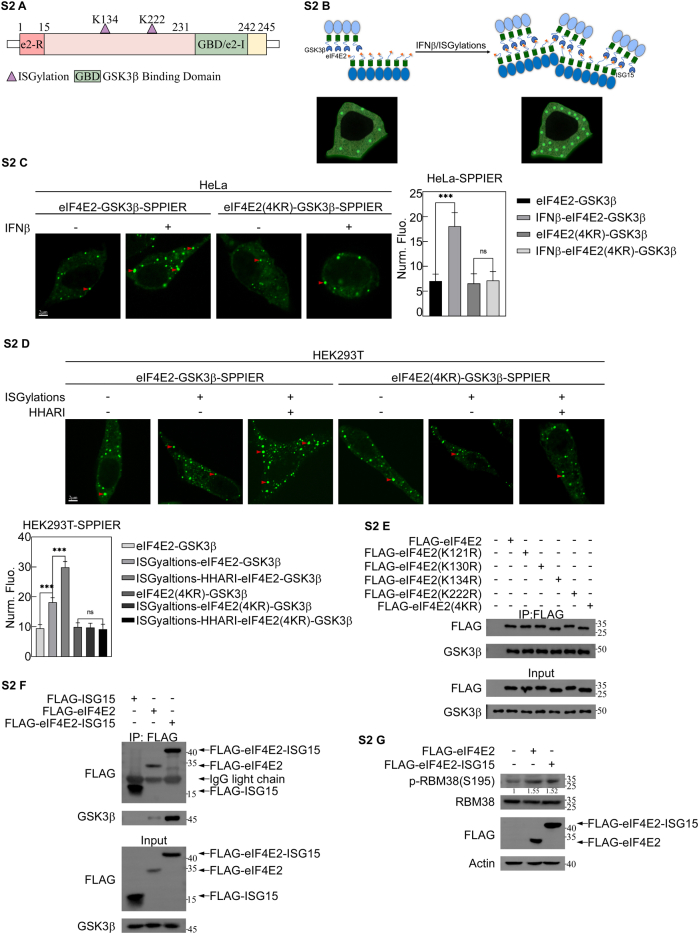


Our previous studies established that eIF4E2 directly interacts with GSK3β and specifically potentiates its S/T-P kinase activity (5). Notably, the key ISGylation site K222 of eIF4E2 is spatially adjacent to its GSK3β-binding domain (residues 231–242), suggesting potential implications for this interaction (Fig. S2*A*).

To elucidate the effect of ISGylation on the interaction between eIF4E2 and GSK3β, we utilized split-pool protein-interaction reporter (SPPIER), a phase separation-based protein–protein interaction detection system (23) (Fig. S2*B*). SPPIER conveniently allows for the comparison of the effects of modifications on protein–protein interactions (23). We found that treating cells with IFNβ for 48 h led to the robust formation of EGFP-positive condensates, indicating enhanced eIF4E2–GSK3β interaction (Fig. S2*C*). Extending IFNβ treatment, a well-established inducer of ISGylation, significantly activated endogenous ISGylation, including the ISGylation of eIF4E2. However, IFNβ treatment failed to augment the interaction between eIF4E2(4KR) and GSK3β, indicating that IFNβ effect on eIF4E2–GSK3β interaction is ISGylation-dependent (Fig. S2*C*). Furthermore, overexpression of ISGylation system comprising UBE1L, UBCH8, and ISG15, enhanced EGFP condensate formation. HHARI coexpression further potentiated this effect. Importantly, the ISGylation system have no effect on the interaction of eIF4E2(4KR) and GSK3β (Fig. S2*D*). To exclude potential structural perturbations caused by lysine mutations, we systematically characterized the effects of individual lysine-to-arginine mutants (K121R, K130R, K134R, K222R) and the 4KR mutant on the interaction between eIF4E2 and GSK3β. FLAG immunoprecipitation (IP) assays demonstrated that all mutants, including eIF4E2-4KR, maintained their interaction with GSK3β, indicating that these mutations do not disrupt the GSK3β-binding domain (Fig. S2*E*). Therefore, these results suggest that ISGylation of eIF4E2 significantly promotes its interaction with GSK3β.

**Figure 4:**
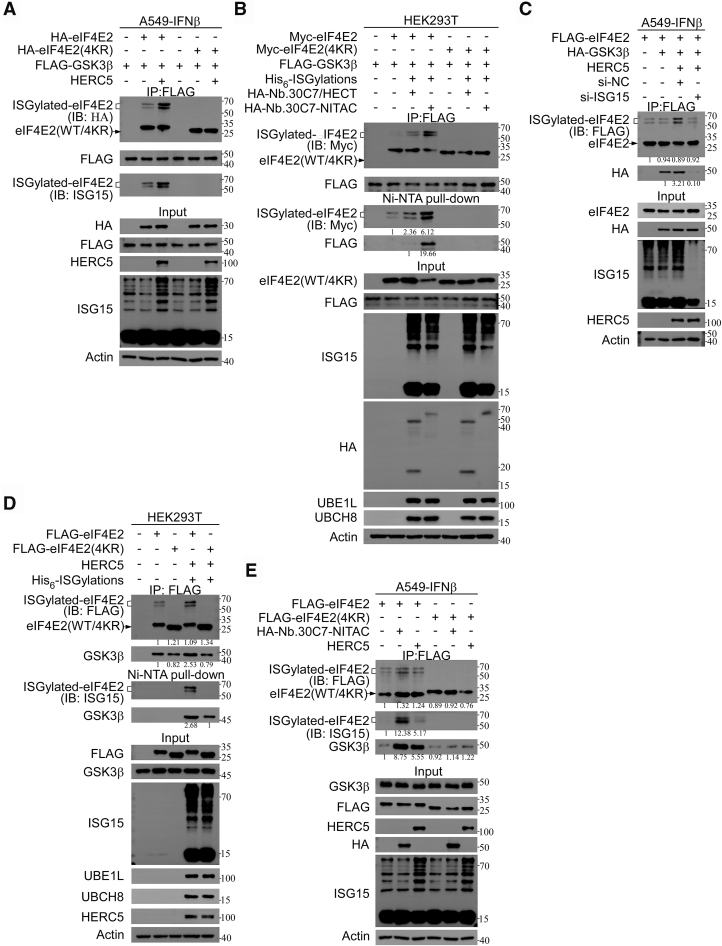
**ISGylation of eIF4E2 enhances its interaction with GSK3β.***A* and *B*, GSK3β interacts with ISGylated eIF4E2. *A*, A549 cells were transfected with plasmids expressing HA-eIF4E2(WT), HA-eIF4E2(4KR), FLAG-GSK3β, and HERC5 as indicated for 12 h and then treated with IFNβ (1000 U/ml) for another 36 h. Then, cell extracts were subjected to Co-IP with anti-FLAG antibody, followed by IB analysis. (*B*) HEK293T cells cotransfected components of the His_6_-ISGylation system (UBE1L, UBCH8, and His_6_-ISG15), plasmids expressing Myc-eIF4E2(WT/4KR), FLAG-GSK3β, HA-Nb.30C7/HA-HECT, and HA-Nb.30C7-NITAC as indicated for 48 h, and then cell extracts were subjected to Co-IP with anti-FLAG antibody and Ni-NTA pull down to precipitate His_6_-ISG15 and its complexes, followed by IB analysis. *C*, depletion of ISG15 inhibits GSK3β from interacting with ISGylated eIF4E2. A549 cells were first transfected with siRNA to knock down the ISG15. After 24 h, plasmids expressing FLAG-eIF4E2, HA-GSK3β, and HERC5 were transfected into the cells, and simultaneously, the cells treated with IFNβ (1000 U/ml) for 36 h. Cell extracts were subjected to co-IP with anti-FLAG antibody, followed by IB analysis. *D* and *E*, ISGylation of eIF4E2 enhances its interaction with GSK3β. *D*, HEK293T cells cotransfected components of the His_6_-ISGylation system (UBE1L, UBCH8, and His_6_-ISG15), plasmids expressing FLAG-eIF4E2(WT/4KR) and HERC5 as indicated for 48 h, and then cell extracts were subjected to co-IP with anti-FLAG antibody and Ni-NTA pull down to precipitate His_6_-ISG15 and its complexes, followed by IB analysis. *E*, A549 cells were transfected with plasmids expressing FLAG-eIF4E2(WT/4KR), HERC5, and HA-Nb.30C7-NITAC as indicated for 12 h and then treated with IFNβ (1000 U/ml) for another 36 h. Then, cell extracts were subjected to co-IP with anti-FLAG antibody, followed by IB analysis. Co-IP, coimmunoprecipitation; eIF4E2, eukaryotic translation initiation factor 4E2; GSK3β, glycogen synthase kinase 3β; IB, immunoblot; IFNβ, interferon β; NITAC, Nanobody-based ISGylation Targeting Chimera.

Consistently, FLAG IP showed that GSK3β interacts with ISGylated eIF4E2 (Fig. 4*A*). FLAG IP and nickel-nitrilotriacetic acid (Ni-NTA) assays based on His_6_-ISGylation system also revealed a specific interaction between GSK3β and ISGylated eIF4E2 (Fig. 4*B*). Importantly, HERC5 expression further promoted the ISGylation of eIF4E2, which interacts with GSK3β (Fig. 4*C*). However, *ISG15* depletion markedly attenuated this interaction, thereby highlighting the critical role of ISGylation in facilitating the interaction of eIF4E2 and GSK3β (Fig. 4*C*).

Furthermore, we conducted a series of coexpression experiments in HEK293T cells. Cells were transfected with FLAG-eIF4E2(WT) or FLAG-eIF4E2(4KR) in the absence and presence of the His_6_-ISGylation system and HERC5. Subsequent FLAG coimmunoprecipitation, Ni-NTA pulldown, and GSK3β immunoblotting revealed that coexpression of the His_6_-ISGylation system and HERC5 significantly enhanced the interaction between FLAG-eIF4E2(WT) and GSK3β. In contrast, FLAG-eIF4E2(4KR) exhibited minimal interaction with GSK3β under identical experimental conditions (Fig. 4*D*), unequivocally demonstrating the pivotal role of ISGylation in promoting protein–protein interactions.

Furthermore, FLAG IP showed that the expression of Nb.30C7-NITAC significantly augmented the interaction between FLAG-eIF4E2(WT) and GSK3β in A549 cells treated with IFNβ (Fig. 4*E*). However, Nb.30C7-NITAC had no effect on the interaction between FLAG-eIF4E2(4KR) and GSK3β (Fig. 4*E*). Therefore, we demonstrated that ISGylation facilitates the eIF4E2–GSK3β interaction, and that NITAC can specifically activate this interaction.

### ISGylation of eIF4E2 inhibits GSK3β′s proline-directed kinase activity

**Figure 5:**
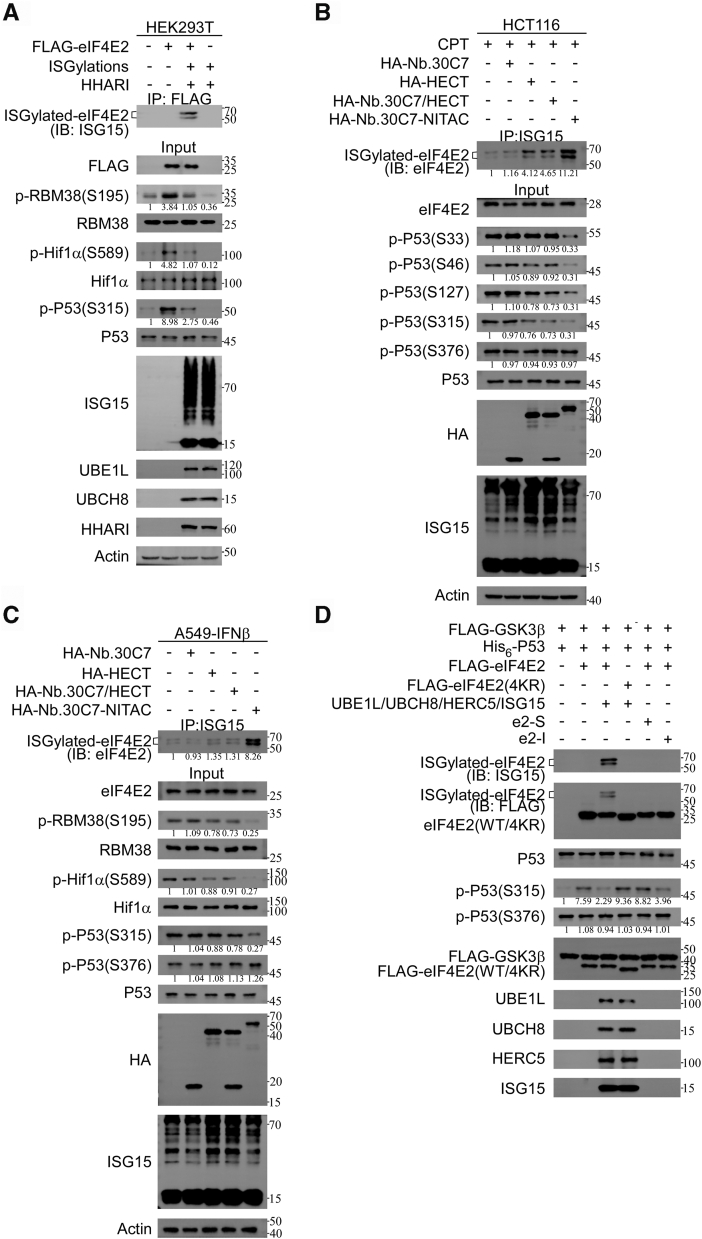
**ISGylation of eIF4E2 inhibits GSK3β kinase activity.***A*, ISGylation inhibited the S/T-P phosphorylation of downstream substrates of the eIF4E2–GSK3β pathway. HEK293T cells cotransfected components of the ISGylation system (UBE1L, UBCH8, and ISG15), plasmids expressing FLAG-eIF4E2and HHARI as indicated for 48 h, and then cell extracts were subjected to co-IP with anti-FLAG antibody, followed by IB analysis. *B* and *C*, ISGylated eIF4E2 inhibited the S/T-P phosphorylation of downstream substrates of the eIF4E2–GSK3β pathway. *B*, HCT116 cell transfected plasmids expressing HA-Nb.30C7, HA-HECT, HA-Nb.30C7/HA-HECT, and HA-Nb.30C7-NITAC as indicated for 24 h and treated with CPT (0.5 μM) for another 24 h. Co-IP with anti-ISG15 and IB analysis of whole-cell extracts. *C*, A549 cell transfected plasmids expressing HA-Nb.30C7, HA-HECT, HA-Nb.30C7/HA-HECT, and HA-Nb.30C7-NITAC as indicated for 12 h and treated with IFNβ (1000 U/ml) for another 36 h. Co-IP with anti-ISG15 and IB analysis of whole-cell extracts. *D*, ISGylated eIF4E2 inhibits GSK3β′s proline-directed kinase activity. E3 ligase HERC5, E2 conjugating enzyme UBE1L, and UBCH8 protein were synthesized by *in vitro* transcription and translation. *In vitro* ISGylation assays were conducted in the presence of ISG15, E1, E2, HERC5, and FLAG-eIF4E2. FLAG-GSK3β, FLAG-eIF4E2, and FLAG-eIF4E2(4KR) proteins were overexpressed and purified from HEK293T cells. His_6_-tagged p53 and ISG15 were expressed and purified from *Escherichia coli*. eIF4E2, HERC5, UBCH8, UBE1L, and ISG15 were incubated in an ATP-containing buffer at 37 °C for 1 h to induce ISGylation of eIF4E2. After the reaction, the resulting components were combined with GSK3β and p53 in an ATP-containing buffer and incubated at 30 °C for 1 h to perform the *in vitro* kinase assay, followed by IB analysis. Co-IP, coimmunoprecipitation; CPT, camptothecin; IB, immunoblot; eIF4E2, eukaryotic translation initiation factor 4E2; GSK3β, glycogen synthase kinase 3β; NITAC, Nanobody-based ISGylation Targeting Chimera; S/T-P, proline-directed serine/threonine.

Translation initiating factor eIF4E2 regulates GSK3β-mediated S/T-P through direct protein–protein interactions, subsequently modulating the phosphorylation status of downstream substrates, including RBM38, HIF1α, and p53 (5). Consistent to this previous report, overexpressed FLAG-tagged eIF4E2 led to a significant activation of S/T-P phosphorylation, including RBM38 (S195), HIF1α (S589), and p53 (S315) in HEK293T cells (Fig. 5*A*). Interestingly, overexpression of the ISGylation system components and the HHARI E3 ligase significantly suppressed the S/T-P phosphorylation of these downstream substrates (Fig. 5*A*), indicating that increased overall ISGylation levels inhibit GSK3β proline-directed kinase activity.

To verify whether the changes in GSK3β proline-directed kinase activity are specifically associated with eIF4E2 ISGylation, we employed eIF4E2 targeting NITAC in HCT116 cells. Consistent to previous research (5, 24), camptothecin-induced DNA damage triggered ISGylation and multisite S/T-P phosphorylation of p53 at sites such as Ser33, Ser46, Ser127, and Ser315, as well as a non–S/T-P at Ser376. Intriguingly, NITAC treatment significantly suppressed the phosphorylation of Ser33, Ser46, Ser127, and Ser315 (Fig. 5*B*). In contrast, NITAC treatment did not affect p53-Ser376 phosphorylation (Fig. 5*B*). These observations were further validated in A549 cells treated with IFNβ through ectopic expression of HECT or NITAC (Fig. 5*C*).

*In vitro* kinase assay was performed to further establish a definitive causal relationship between eIF4E2 ISGylation and GSK3β′s proline-directed kinase activity (25). We reconstituted the ISGylation system *in vitro* using purified components (26), including HERC5, E2 enzyme UBCH8, E1 enzyme UBE1L, and ISG15 to mediate the ISGylation of eIF4E2. The results showed that eIF4E2(WT) underwent ISGylation in the presence of ISGylation system (UBE1L, UBCH8, and ISG15) and HERC5, while eIF4E2(4KR) remained unmodified (Fig. 5*D*). Then, *in vitro* kinase assay showed that both eIF4E2(WT) and 4KR mutant further enhanced GSK3β-mediated S/T-P phosphorylation of p53-Ser315, but showed no effect on the non–S/T-P of p53-Ser376 (Fig. 5*D*). Notably, reconstituted ISGylation of eIF4E2 specifically attenuated GSK3β proline-directed kinase activity, but showed no effect on the action of eIF4E2(4KR) (Fig. 5*D*). *In vitro* kinase assay also showed that the specific inhibitory peptide e2-I, which interferes with the eIF4E2–GSK3β interaction, inhibited the eIF4E2–GSK3β pathway, reducing p53-Ser315 phosphorylation without affecting p53-Ser376 phosphorylation (Fig. 5*D*). As a control, a scrambled peptide (e2-S) had no impact on the eIF4E2–GSK3β pathway.

ISG15 has generally been directly fused to target proteins for functional studies. As previously demonstrated, this strategy revealed that eIF4E2 ISGylation may increase its 5′ cap-binding potential (15). Our results showed that the Flag-eIF4E2-ISG15 fusion protein exhibited significantly stronger binding to GSK3β than Flag-ISG15 and Flag-eIF4E2 alone (Fig. S2*F*), recapitulating the effect of ISGylated eIF4E2 as observed under the ISGylation system. However, the overexpression of eIF4E2 fused with ISG15 failed to alter the phosphorylation status of RBM38 at residue S195 (Fig. S2*G*), indicating functional differences of fusion proteins with site-specific ISGylation.

These results suggest that eIF4E2 ISGylation enhances its interaction with GSK3β, but this interaction exerts negative effects on GSK3β′s proline-directed kinase activity rather than activating it. Additionally, these results indicate that using NITAC to achieve site-specific ISGylation more accurately reflects the functional effects of ISGylation compared to the fusion strategy.

### NITAC-enabled eIF4E2 ISGylation provides cytoprotection post-OGD/R

**Figure 6:**
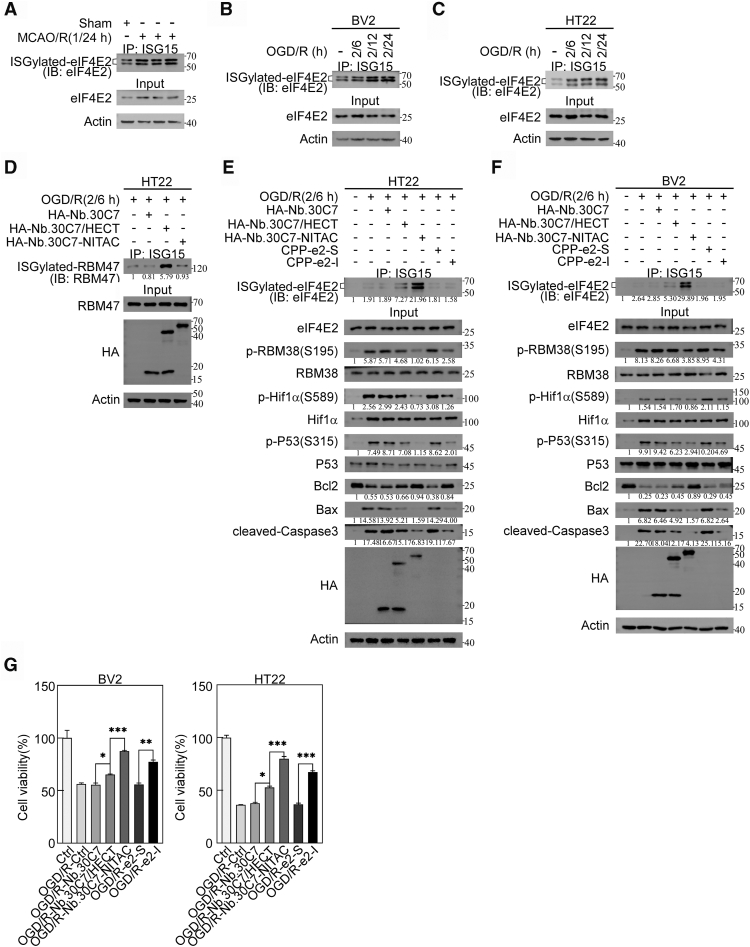
**NITAC-enabled eIF4E2 ISGylation provides cytoprotection post-OGD/R.***A*, eIF4E2 ISGylation is activated in the MCAO/R mouse model. IB analysis was performed on cortical tissues from MCAO/R and Sham group mice. *B* and *C*, eIF4E2 ISGylation is activated in the OGD/R cell model. BV2 (*B*) and HT22 (*C*) cells were subjected to 2 h of OGD followed by 6, 12, or 24 h of reoxygenation. Cell lysates were analyzed by IB. *D*, specificity verification of NITAC. HT22 cells were subjected to 2 h of OGD followed by 6 h of reoxygenation. Cell samples were analyzed by IB. *E*-*G*, NITAC-mediated eIF4E2 ISGylation inhibits GSK3β′s proline-directed kinase activity, thereby reducing apoptosis and promoting cell survival under OGD/R conditions. Data were expressed as mean ± SD. Statistical significance was determined by one-way mean ANOVA (n = 3 per group, ∗: *p <* 0.05, ∗∗: *p <* 0.01, ∗∗∗: *p <* 0.001, and ∗∗∗∗: *p* < 0.0001). eIF4E2, eukaryotic translation initiation factor 4E2; GSK3β, glycogen synthase kinase 3β; IB, immunoblot; MCAO/R, middle cerebral artery occlusion/reperfusion; NITAC, Nanobody-based ISGylation Targeting Chimera; OGD, oxygen-glucose deprivation.

S/T-P is widely present in the nervous system, but its significance remains poorly understood. Therefore, the functional implications of eIF4E2 ISGylation and its regulation of GSK3β′s proline-directed kinase activity warrant further investigation. In our previous research on ischemic stroke, we discovered that eIF4E2 ISGylation may play a role in its pathological processes. We used a murine middle cerebral artery occlusion/reperfusion (MCAO/R) model to induce stroke, and post-MCAO/R analysis revealed significantly elevated eIF4E2 ISGylation in the ipsilateral cortex compared to sham controls, while total eIF4E2 expression remained unchanged (Fig. 6*A*). To mechanistically dissect this phenomenon *in vitro*, we employed two central nervous system-relevant cell models: microglial BV2 cells (critical mediators of neuroinflammation in stroke) and neuronal HT22 cells (representing hippocampal neurons vulnerable to ischemic injury). Consistently, OGD/reoxygenation (OGD/R) treatment, mimicking ischemic conditions, significantly enhanced eIF4E2 ISGylation in both microglial BV2 (Fig. 6*B*) and neuronal HT22 (Fig. 6*C*) cells.

The application of eIF4E2-targeting NITAC may help elucidate its function in stroke. We found that the expression of both Nb.30C7/HECT and Nb.30C7-NITAC significantly increased eIF4E2 ISGylation under OGD/R (2/6 h) conditions in HT22 and BV2 cells (Fig. 6, *E* and *F*), with Nb.30C7-NITAC showing a more pronounced effect. It is known that the RNA-binding protein RBM47 undergoes ISGylation (27). Importantly, Nb.30C7/HECT significantly enhanced RBM47 ISGylation, while the eIF4E2-specific Nb.30C7-NITAC showed no additional impact on RBM47 ISGylation, thereby demonstrating the target specificity of NITAC (Fig. 6*D*).

Notably, Nb.30C7-NITAC significantly suppressed phosphorylation of RBM38 (Ser195), Hif1α (Ser589), and p53 (Ser315), indicating that NITAC-mediated eIF4E2 ISGylation effectively inhibits GSK3β proline-directed kinase activity under OGD/R (2/6 h) conditions in HT22 and BV2 cells (Fig. 6, *E* and *F*). e2-I peptide also suppressed these phosphorylations, indicating that eIF4E2–GSK3β pathway and GSK3β′s proline-directed kinase activity is activated under OGD/R (2/6 h) conditions (Fig. 6, *E* and *F*). Given the pivotal role of apoptotic cell death in ischemic stroke pathology, particularly under OGD/R conditions (28), we analyzed the impact of NITAC-mediated eIF4E2 ISGylation on apoptosis (as indicated by Bcl2, Bax, and cleaved-caspase3 levels) following OGD/R (2/6 h) treatment. The expression of eIF4E2-targeting NITAC markedly attenuated proapoptotic proteins (Bax and cleaved-caspase3), while simultaneously elevating anti-apoptotic Bcl2 expression in both HT22 (Fig. 6*E*) and BV2 cells (Fig. 6*F*), indicating efficient suppression of OGD/R-induced apoptosis. Consistently, cell viability assays (CCK8) revealed expression of eIF4E2-targeting NITAC significantly improved survival of BV2 (Fig. 6*G*, left) and HT22 cells (Fig. 6*G*, right) following OGD/R exposure, compared to the expression of Nb.30C7/NITAC. Furthermore, e2-I treatment also inhibited apoptosis and promoted cell survival (Fig. 6, *G*–*F*), also indicating the protective effect of inhibiting GSK3β proline-directed kinase activity.

**Supporting Figure S3:**
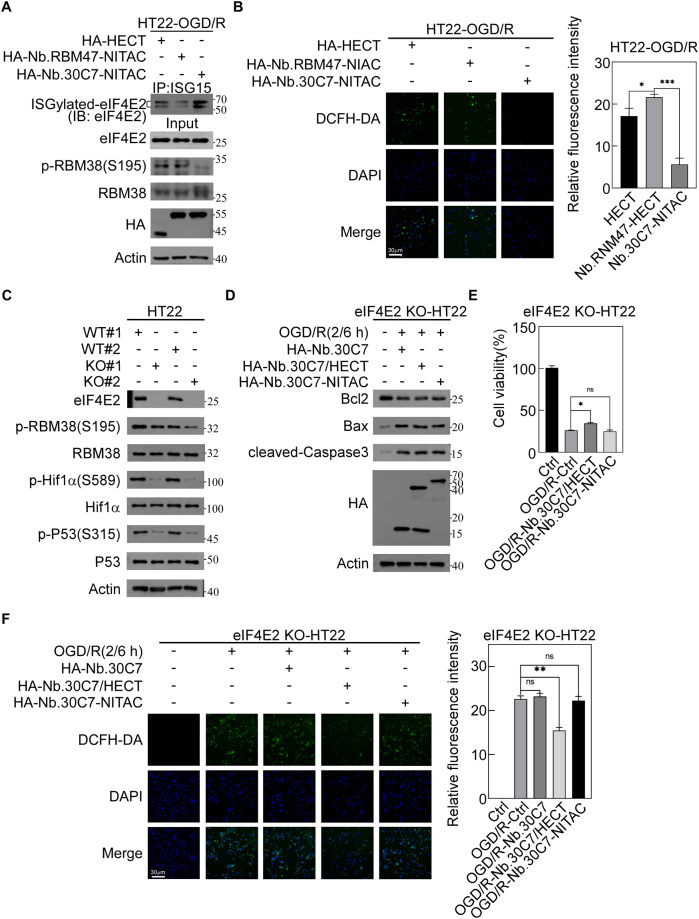


To further demonstrate the cytoprotective effects of NITAC depend on eIF4E2 ISGylation, we recently constructed RBM47-targeting NITAC using its specific nanobody (27). Overexpression of Nb.RBM47-NITAC in HT22 neuronal cells specifically mediated RBM47 ISGylation. However, IP with an anti-ISG15 antibody revealed that expression of RBM47-targeting NITAC did not induce eIF4E2 ISGylation (Fig. S3*A*) nor did it regulate RBM38 S/T-P phosphorylation (Fig. S3*A*). Crucially, unlike eIF4E2-targeting NITAC, overexpression of Nb.RBM47-NITAC in HT22 neurons failed to reduce reactive oxygen species (ROS) accumulation following OGD/reoxygenation (OGD/R) injury (Fig. S3*B*). To directly test whether NITAC's cytoprotective effects require eIF4E2, we generated eIF4E2 KO HT22 cell lines using CRISPR/Cas9 technology (Fig. S3*C*). Consistent with previous studies (5), eIF4E2 deletion significantly reduced the phosphorylation levels of RBM38 (Ser195), Hif1α (Ser589), and p53 (Ser315). Interestingly, overexpression of Nb.30C7-NITAC in eIF4E2-KO HT22 cells did not significantly alter the expression of apoptotic markers under OGD/R (2/6 h) conditions (Bax, cleaved-caspase3, and Bcl2) (Fig. S3*D*). Similarly, cell viability assays revealed no significant improvement in the survival of eIF4E2-null HT22 cells upon their expression post-OGD/R (2/6 h) (Fig. S3*E*). In comparison, the expression of HECT had a lesser effect on both apoptosis and cell survival in HT22 cells. These findings demonstrate that eIF4E2 ISGylation is a critical mediator of NITAC-induced cytoprotection following ischemic injury.

### NITAC-enabled eIF4E2 ISGylation inhibits oxidative stress and inflammation post-OGD/R

**Figure 7:**
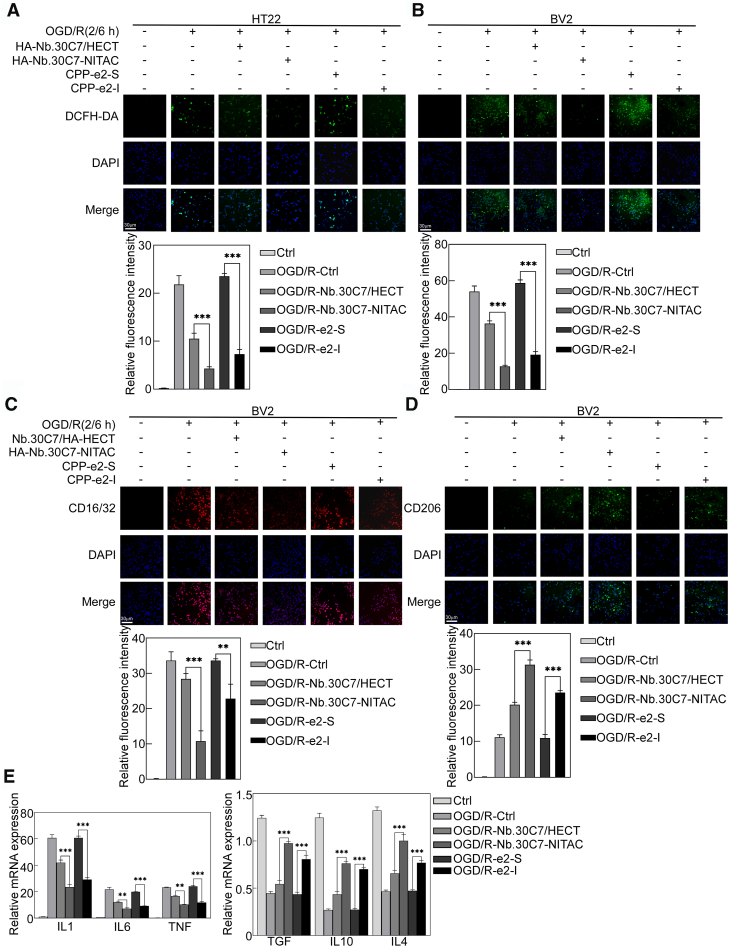
**NITAC-enabled eIF4E2 ISGylation inhibits oxidative stress and inflammation post-OGD/R.***A* and *B*, NITAC-mediated eIF4E2 ISGylation reduced ROS generation in HT22 (*A*) and BV2 (*B*) cells after OGD/R. ROS levels were measured using DCFH-DA staining, followed by fluorescence microscopy. The scale bar represents 30 μm. Data are presented as the mean ± SD. Statistical significance was determined by one-way ANOVA (n = 3 for each group, ∗: *p <* 0.05, ∗∗: *p <* 0.01, ∗∗∗: *p <* 0.001, and ∗∗∗∗: *p* < 0.0001) (*shown in the bottom panel*).*C* and *D*, NITAC-mediated eIF4E2 ISGylation reversed phenotypic switching in BV2 cells following OGD/R. BV2 cells were treated as indicated, and M1 marker CD16/32 (*C*) and M2 marker CD206 (*D*) expression were analyzed by fluorescence microscopy. The scale bar represents 30 μm. Data are presented as the mean ± SD. Statistical significance was determined by one-way ANOVA (n = 3 for each group, ∗: *p* < 0.05, ∗∗: *p* < 0.01, ∗∗∗: *p* < 0.001, and ∗∗∗∗: *p* < 0.0001) (*shown in the bottom panel*).*E*, NITAC-mediated eIF4E2 ISGylation inhibited the expression of proinflammatory genes and promoted the expression of anti-inflammatory genes after OGD/R. BV2 cells were treated as indicated, and expression levels of proinflammatory genes (*IL-1β*, *IL-6*, *TNF-α*) and anti-inflammatory genes (*IL-4*, *IL-10*, *TGF-β*) were assessed using qPCR. Data are presented as the mean ± SD. Statistical significance was determined by one-way ANOVA (n = 3 for each group, ∗: *p* < 0.05, ∗∗: *p* < 0.01, ∗∗∗: *p* < 0.001, and ∗∗∗∗: *p* < 0.0001). eIF4E2, eukaryotic translation initiation factor 4E2; IB, immunoblot; NITAC, Nanobody-based ISGylation Targeting Chimera; ROS, reactive oxygen species.

To investigate the mechanistic basis of NITAC-mediated cytoprotection, we examined its effects on oxidative stress and inflammatory responses, two critical pathological processes in OGD/R-induced neuronal injury (28). Oxidative stress was assessed by measuring intracellular ROS levels using 2′,7′-dichlorofluorescin diacetate fluorescence. OGD/R (2/6 h) exposure significantly elevated ROS levels in both BV2 microglial cells and HT22 neurons. While both HECT and NITAC expression attenuated ROS accumulation (Fig. 7, *A* and *B*), NITAC exhibited superior antioxidant efficacy. e2-I, a selective inhibitor of GSK3β S/T-P kinase activity, demonstrated similar ROS-scavenging effects. Notably, this protective effect of NITAC was completely abolished in eIF4E2-deficient HT22 cells (Fig. S3*D*).

Given the critical role of microglial activation in postischemic injury progression (29), we next examined microglial polarization states using established phenotypic markers. Under basal conditions, BV2 microglia maintained a quiescent state, as evidenced by low expression of both M1 (CD16/32) and M2 (CD206) markers (Fig. 7, *C* and *D*). OGD/R (2/6 h) exposure induced significant upregulation of both markers, indicating enhanced inflammatory activation. The expression of eIF4E2-targeting NITAC markedly suppressed CD16/32 expression, while concomitantly enhancing CD206 expression compared to HECT overexpression, suggesting a preferential polarization toward an anti-inflammatory M2 phenotype. Real-time quantitative PCR (qPCR) analysis revealed that OGD/R significantly upregulated proinflammatory M1 markers (interleukin [IL]*-1β*, *IL-6*, and tumor necrosis factor*-α*), while suppressing anti-inflammatory M2 markers (*IL-4*, *IL-10*, and transforming growth factor*-β*). The expression of eIF4E2-targeting NITAC effectively reversed this inflammatory signature, simultaneously downregulating proinflammatory cytokines and enhancing anti-inflammatory mediator expression (Fig. 7*E*). The GSK3β inhibitor e2-I showed similar immunomodulatory effects on BV2 cells under OGD/R conditions, similarly revealing the protective role of GSK3β S/T-P kinase activity in ischemic stroke. Collectively, these findings demonstrate that eIF4E2-targeting NITAC confers cytoprotection through attenuating OGD/R-induced oxidative stress and inflammatory responses in both microglial and neuronal cells.

## Discussion

eIF4E2, an eIF4E homolog (also known as 4EHP), competes with eIF4E for binding to the mRNA 5′ cap structure, thereby modulating translation initiation and repressing cap-dependent translation (30). Under hypoxic conditions, eIF4E2 facilitates selective translation initiation of specific mRNA transcripts (4). Previous studies demonstrated that the E3 ligase HHARI catalyzes ISGylation of eIF4E2 at lysine residues K134 and K222, enhancing its cap-binding affinity to regulate innate immunity (15). Intriguingly, eIF4E2 also modulates GSK3β′s proline-directed kinase activity, participating in hypoxia-induced signal transduction (5). While proteins such as FRAT-1, FRAT-2, and LRP6 interact with and regulate GSK3β kinase activity (31), eIF4E2 shares a conserved GSK3β-binding motif with these proteins. Notably, the K222 ISGylation site of eIF4E2 is proximally located to its GSK3β-binding domain (amino acids 231–242) (5). Interestingly, the interaction between eIF4E2 and GSK3β activates the proline-directed kinase activity of GSK3β (5). S-nitrosylation of GSK3β at Cys317 disrupts this interaction and subsequently represses its proline-directed kinase activity (5). In this study, we developed a novel NITAC tool that specifically targets eIF4E2, enhancing its ISGylation. The use of this NITAC tool indicates that eIF4E2 ISGylation acts as a novel regulatory mechanism. Specifically, it enhances the interaction between eIF4E2 and GSK3β, but paradoxically suppresses GSK3β′s proline-directed kinase activity.

The development of PROTACs has shown that small molecules or nanobodies can direct E3 ligases to achieve ubiquitination of target proteins, subsequently mediating protein degradation (17, 18). The ISGylation process is similar to ubiquitination, involving a cascade of E1, E2, and E3 ligases to modify target proteins with ISG15 (32). Unlike ubiquitination, ISGylation components are typically low in normal cells and require activation by interferons or stress signals (6). Common activation methods include overexpression of the ISGylation system or treatment with IFNβ (16). In our study, we activated eIF4E2 ISGylation by introducing NITAC in HEK293T cells overexpressing the ISGylation system (E1, E2, and ISG15) or in A549 and HeLa cells treated with IFNβ. Research shows that NITAC, dependent on eIF4E2-targeting nanobodies and HECT (the catalytic E3 ligase domain from HERC5) fusion, effectively activates eIF4E2 ISGylation (Fig. 3). While overexpressing HECT can also activate eIF4E2 ISGylation, it affects overall ISGylation, interfering with eIF4E2 studies (Figs 3 and S1, *C* and *D*). eIF4E2-targeting NITAC exhibits off-target effects on total protein ISGylation in HEK293T cells overexpressing the ISGylation system (Fig. S1, *C* and *D*). Notably, this NITAC shows better specificity in A549 and HeLa cells treated with IFNβ (Fig. 3, *B*–*G*). Further optimization of nanobodies to enhance their affinity could improve NITAC’s targeted modification capabilities and specificity.

Specifically, we found that high-level expression of the HERC5 HECT domain, particularly in cells with robust ISGylation capacity such as HEK293T overexpressing the ISGylation machinery, promotes broad protein ISGylation, including modification of endogenous eIF4E2 (Fig. S1*B*). In contrast, overexpression of HECT in IFNβ-treated HeLa cells for 24 h, which display low basal ISGylation activity, did not induce global ISGylation or eIF4E2-specific modification. These observations indicate that HECT-mediated ISGylation is highly dependent on both the expression level of the catalytic domain and the intrinsic ISGylation potential of the host cell. This finding aligns with prior studies identifying the HECT domain as the core catalytic unit, while the RCC1-like domain confers substrate specificity through polysome association (21, 33). Our NITAC system emulates this natural targeting strategy by replacing the substrate-recognition domain with a nanobody, thereby enabling spatially confined and substrate-selective ISGylation. As such, the current NITAC platform provides a valuable tool for achieving precise, site-specific protein modification in living cells. Moreover, combining ISG15 IP with quantitative mass spectrometry to profile ISGylated substrates across diverse cell types may provide a systematic means to evaluate both the strengths and limitations of the NITAC system. Although existing mass spectrometry–based ISGylome profiling approaches, including ISG15 enrichment and diGLY remnant capture, support high-throughput and site-resolved analysis, their performance remains strongly influenced by ISGylation abundance, enrichment specificity, and interference from related ubiquitin-like modifiers such as ubiquitin and NEDD8 (34, 35, 36). To address these challenges, we are actively planning to integrate NITAC-mediated substrate targeting with proteomic analysis in future studies, aiming to identify novel, physiologically relevant ISGylation events.

Previously established approaches for studying specific protein ISGylation primarily utilized chimeric proteins incorporating ISG15 at either the amino (N) or carboxyl (C) terminus of target proteins (37). These fusion constructs theoretically recapitulate constitutive protein modification, particularly when native modification sites are proximally located to protein termini (37). Both N- and C-terminal ISG15 fusion to eIF4E2 effectively enhance its cap structure-binding activity (15). Similarly, C-terminal ISG15-eIF4E2 fusion enhanced the interaction between GSK3β and eIF4E2, similar to the effects observed with NITAC-mediated ISGylation (Fig. 5*D*). However, while NITAC-induced ISGylation significantly attenuated GSK3β′s proline-directed kinase activity, the C-terminal ISG15-eIF4E2 fusion showed no appreciable effect on GSK3β′s proline-directed kinase activity. This suggests that site-specific modification and terminal fusion approaches have distinct functional consequences (Fig. 5*E*). GSK3β′s kinase activity is regulated by various complex mechanisms, including diverse modulation by interacting proteins. For example, despite sharing 70% sequence homology, FRAT-1 and FRAT-2 exert opposing effects on GSK3β activity (38). Although these proteins are homologous and regulate GSK3β through a conserved GSK3β-binding domain, they produce distinct outcomes. eIF4E2's ISGylation enhances its interaction with GSK3β, but likely induces complex conformational changes in eIF4E2, potentially affecting the architecture of GSK3β′s catalytic domain and subsequently inhibiting its proline-directed kinase activity. Further structural analyses are warranted to elucidate these molecular mechanisms. We believe that NITAC enables site-specific modification, making it a superior approach for investigating ISGylation effects. Unlike ISG15 fusion, NITAC mediates eIF4E2 ISGylation, providing a more accurate representation of physiological conditions.

GSK3β has emerged as a potential therapeutic target in ischemic stroke through its regulation of oxidative stress and inflammatory processes (39). Its dual kinase activities, particularly S/T-P–directed phosphorylation, represent fundamental signaling mechanisms in cellular processes including proliferation and differentiation (40). Dysregulation of S/T-P phosphorylation has been implicated in various pathological conditions, including stroke, neurodegenerative disorders, and cancer (28). NITAC-mediated eIF4E2 ISGylation downregulates GSK3β proline-directed kinase activity, providing significant cytoprotective benefits in neurons and microglial cells after OGD/reoxygenation (OGD/R) injury (Figs. 6, *E*–*G*, and 7). Similarly, we demonstrated that treating with the peptide e2-I, which inhibits eIF4E2–GSK3β interaction, specifically suppresses GSK3β proline-directed kinase activity and exerts cytoprotective effects under OGD/R stress conditions.

Accumulating evidence suggests that ISGylation plays a crucial role in endogenous neuroprotective responses following ischemic injury. Genetic ablation of either ISG15 or its E1 activating enzyme UBE1L exacerbated ischemic brain injury in murine MCAO models (41, 42). Diminished USP18 expression disrupts protein ISGylation, leading to neuronal damage, blood-brain barrier dysfunction, and neurological deficits (43). Recent studies have documented elevated protein ISGylation in cortical regions following transient ischemic attacks (44). ISGylation-deficient mice exhibiting enhanced susceptibility to ischemic brain injury (42). Global cerebral ischemia models have demonstrated widespread activation of brain protein ISGylation, particularly prominent in hippocampal regions (8). However, these studies collectively highlight the significance of ISGylation in the nervous system following stroke injury, but given the diversity of ISG15 substrates, the specific roles of substrate-specific ISGylation remain unclear. Our study presents the first demonstration of the cytoprotective effects mediated by the ISGylation of specific proteins. Additionally, enhanced eIF4E2 ISGylation may further exert cytoprotective effects by regulating the translation of specific target transcripts, which still requires further investigation using the NITAC tool. While the cytoprotective role of the eIF4E2–GSK3β axis is established *in vitro*, its neuroprotective potential warrants *in vivo* validation.

In summary, this study constructs an effective selective tool, NITAC, which specifically activates eIF4E ISGylation and demonstrates that eIF4E ISGylation inhibits GSK3β proline-directed kinase activity. Using these methods, we found that eIF4E2 ISGylation provides significant cytoprotection in neurons and microglial cells after OGD/reoxygenation (OGD/R) stress, highlighting the therapeutic potential of targeting protein ISGylation in modulating intracellular signaling in ischemic stroke.

## Experimental procedures

### Reagent or resource

The used reagent and resource were listed in Table S1.

The phospho-specific antibodies p-RBM38 was generated by immunizing rabbits with the synthetic phospho-peptide span serine195 [^187^YDQYPYAAS(p)PAT^198^] (5). The RBM38 antibodies were generated by immunizing rabbits with the peptide [^195^SPATAASFVGYS^206^] (5). The phospho-specific p-HIF1α(S589) antibodies were generated by immunizing rabbits with the synthetic phospho-peptide span serine 589 [^584^ESSSAS(p)PES^592^] (5). The RBM47 antibodies were generated by immunizing rabbits with the peptide [^565^GGYAGYIPQAFPAALQ^580^] (27).

### Plasmids

Plasmid constructs for the expression of UBE1L, UBCH8, HERC5, HERC5(C994A), ISG15, and 3xFLAG-tagged ISG15 were previously reported (27) and were purchased from Addgene (Addgene Inc). Similarly, constructs for expressing the SPPIER plasmid (EGFP-eIF4E2-HOTag3-T2A-GSK3β-HOTag6), GST-tagged eIF4E2, HA-tagged eIF4E2, 3xFLAG-tagged eIF4E2, HA and 3xFLAG-tagged GSK3β, His_6_-tagged Nb.BV025, p53, and Hif1α were described in a prior publication (5). We generated additional pcDNA3-based ISG15 plasmids encoding a His_6_ (HHHHHH) tag at the N terminus of ISG15 or ISG15-fused eIF4E2. The eIF4E2 open reading frame and its variants (K121, K130R, K134R, K222R, and 4KR) were subsequently subcloned into pcDNA3 vectors containing amino-terminal epitope tags, including Myc (EQKLISEEDL), 3xFLAG (MDYKDHDGDYKDHDIDYKDDDDK), HA (YPYDVPDYA) tags, and ISG15 (LRLRGG). The HHARI ORF and its variants were subsequently subcloned into pcDNA3 vectors containing HA (YPYDVPDYA) tags.

The used PCR primers were listed in Table S2.

### SPPIER assay

SPPIER (separation of phases-based protein interaction reporter) assay is based on multivalent protein-protein interaction-induced protein phase transition, which leads to formation of highly concentrated protein droplets (23, 45). SPPIER was performed as described previously (5) with some modifications. Cells were grown in 35 mm glass-bottom microwell (20 mm) dishes (801001, NEST). HeLa cells were transfected with the constructed SPPIER plasmid (EGFP-eIF4E2 (WT/4KR)-HOTag3-T2A-GSK3β-HOTag6) and treated with IFNβ (1000 units/ml) for 48 h. HEK293T cells were transfected with the constructed SPPIER plasmid, ISGylations system (UBE1L, UBCH8, and ISG15), and HHARI using Lipofectamine 3000 reagent (Invitrogen). The plasmids were cotransfected at a carefully optimized mass ratio of UBE1L: UBCH8: ISG15: HHARI: SPPIER as 1: 1: 2: 2: 2, respectively. Specifically, for a 6-well plate, the total plasmid amount was maintained at 2 μg, with individual plasmid quantities proportionally adjusted to maintain the specified ratio. Transfection was performed following the manufacturer's recommended protocol, and cells were harvested after a 48-h incubation period. Image acquisition is performed using spinning disk confocal microscope (Andor), ImageJ software (National Institutes of Health; available at https://imagej.nih.gov/ij/) was used to process the image.

### Immunoblot and coimmunoprecipitation analysis

Immunoblot (IB) analysis was performed as previously described (46). Cell lysates suspended in SDS-PAGE sample buffer (62.5 mM Tris–HCl, pH 6.8, 2% SDS, 10% glycerol, 0.5% bromophenol blue, 0.5% β-mercaptoethanol) were resolved by SDS-PAGE, transferred to a PVDF membrane, and probed with indicated antibodies. The immunoreactive bands were visualized by the enhanced chemiluminescence (Pierce) and quantified by densitometry with Chemiscope 6000 Exp (Chemi, CHN). IP assay was performed as previously described (46). Briefly, cells were lysed in 0.5% Triton lysis buffer (25 mM Tris pH 7.5, 25 mM NaCl, 0.5% Triton X-100) supplemented with the proteinase inhibitor cocktail (100 mg/ml), followed by incubation with 1 mg of antibody. The immunocomplexes were brought down by protein A/G beads and subjected to IB analysis.

### The construction of a synthetic yeast display nanobody library

The construction of the nanobody library followed the previously described protocol (47). Briefly, the DNA library of nanobodies was constructed by two-step overlap-extension PCR. A set of 10 primers (47) (Tsingke Biotechnology Co., Ltd) were dissolved and mixed to prepare three mixed pools, “mix short,” “mix medium,” and “mix long,” differed in CDR3 region of variable length of 7, 11, or 15 randomized residues respectively. The full-length nanobody DNA product from each pool was mixed in a 1:2:1 M ratio of short/medium/long CDR3 regions, referred to as the nanobody DNA library pool hereafter, recapitulating the length distribution frequencies observed in camelid VHH domains. The nanobody DNA library pool was amplified *via* yeast transformation using primers designed for homologous recombination. The yeast library was created by transformation of yeast with linearized yeast display vector (pNACP) and the PCR insertion products, and *in vivo* homologous recombination occurs between the vector and PCR insert to generate the display plasmid. Five hundred milliliters of *Saccharomyces cerevisiae* cells (EBY100) were grown to A_600_ 1.6 and transformed with 120 μg nanobody insert DNA and 40 μg of pNACP plasmid, and digested with HindⅢ-HF and BamHI-HF (New England BioLabs), using an MicroPulser (Bio-Rad, No. 411BR12986). Prepare 10-fold serially diluted cells from the cell suspension and plate 100 μl of 1/10^6^, 1/10^7^, and 1/10^8^ diluted cells onto selective plates and incubated at 30 °C incubator. Library size is determined from the colony counts and sequencing reactions. Grow remaining cells in selective medium overnight. This is the transformed library. The yeast library was subsequently generated with a capacity of 2.5 × 10^9^.

Initial MACS selections can process a large number of cells. MACS selection enriches clones that bind to antigens and reduces the library diversity to a level manageable by FACS for further processing (48). Incubate the cells with biotinylated antigen (BSA-e2-I), followed by the addition of Miltenyi MACS streptavidin microbeads for further incubation. Process the cells through an LS column under a magnetic field, and elute the beads with SDCAA solution. Transfer the eluted beads and cells into SDCAA medium for cultivation. Once the A600 reaches 0.5 to 1, transfer the cells to SGCAA medium to induce nanobody expression. Collect the expressed yeast library and perform MACS selection again. After two rounds of MACS selection, collect the yeast library for FACS sorting. The peptide used for eIF4E2 nanobody screening was the e2-I/e2-R peptide with the sequence RLLFQNLWKPRL/MNNKFDALKDDDSGD (5). The BSA-fused peptide was diluted in FACS buffer (PBS, pH 7.4, 10 mM EDTA) and preincubated with anti-BSA antibody (mouse, 1:200) for 1 h at room temperature. Following galactose induction of nanobodies, 1 × 10^10^ yeast cells were resuspended in FACS buffer and incubated with the pretreated BSA peptide for 1 h at room temperature, followed by washing with FACS buffer. Yeast displaying nanobodies were incubated with anti-myc antibody, labeled with anti-rabbit IgG-Alexa Fluor 647 (1: 200), and the pretreated BSA peptide was labeled with anti-mouse IgG-FITC (1: 50). Yeast populations exhibiting dual fluorescence of FITC and Alexa Fluor 647 were enriched by flow cytometry, using unlabeled yeast cells as negative controls. Four rounds of flow cytometry screening were conducted with sequential 10-fold decreases in antigen (BSA peptide) concentration, ranging from 1 μM to 10 nM. Post-FACS screening, yeast cells were isolated into single colonies and cultivated as clonal cultures in 96-well microplates. Upon galactose induction for nanobody expression, yeast clones were stained using an FITC-conjugated eIF4E2 peptide (e2-I/e2-R). The specificity and binding affinity of the candidate nanobodies were validated using analytical flow cytometry, confirming the selection of clones with the requisite binding properties.

The following are the protein sequences of the nanobody:

Nb.28E11: MQVQLQESGGGLVQAGGSLRLSCAASGRTFSSYAMGWFRQAPGKEREFVAAISSGGSITNYADSVKGRFTISRDNAKNTVYLQMNSLKPEDTAVYYCAARYHSLYYGYWGQGTQVTVSS

Nb.30C7: MQVQLQESGGGLVQAGGSLRLSCAASGYTFSSNVMGWFRQAPGKEREFVAAINSGGGRTNYADSVKGRFTISRDNAKNTVYLQMNSLKPEDTAVYYCAAYGRANSSYNYGYWGQGTQVTVSS

### The construction of NITAC

HERC5 is an interferon-induced HECT-type E3 protein ligase that comprises an N-terminal RCC1-like domain and a C-terminal HECT domain (residues 681–1024). To alter HERC5’s substrate specificity and achieve targeted modulation of the eIF4E2 protein, we fused Nb.30C7 to the N terminus of the HECT domain *via* a flexible linker (SSSGS), thereby constructing the tool enzyme Nb.30C7-NITAC for the targeted activation of eIF4E2 ISGylation.

The following are the protein sequences of the NITAC (Nb-HECT_681-1024aa_):

Nb.28E11-NITAC: MQVQLQESGGGLVQAGGSLRLSCAASGRTFSSYAMGWFRQAPGKEREFVAAISSGGSITNYADSVKGRFTISRDNAKNTVYLQMNSLKPEDTAVYYCAARYHSLYYGYWGQGTQVTVSSSGSMFDLTVRRNHLIEDVLNQLSQFENEDLRKELWVSFSGEIGYDLGGVKKEFFYCLFAEMIQPEYGMFMYPEGASCMWFPVKPKFEKKRYFFFGVLCGLSLFNCNVANLPFPLALFKKLLDQMPSLEDLKELSPDLGKNLQTLLDDEGDNFEEVFYIHFNVHWDRNDTNLIPNGSSITVNQTNKRDYVSKYINYIFNDSVKAVYEEFRRGFYKMCDEDIIKLFHPEELKDVIVGNTDYDWKTFEKNARYEPGYNSSHPTIVMFWKAFHKLTLEEKKKFLVFLTGTDRLQMKDLNNMKITFCCPESWNERDPIRALTCFSVLFLPKYSTMETVEEALQEAINNNRGFG

Nb.30C7-NITAC: MQVQLQESGGGLVQAGGSLRLSCAASGYTFSSNVMGWFRQAPGKEREFVAAINSGGGRTNYADSVKGRFTISRDNAKNTVYLQMNSLKPEDTAVYYCAAYGRANSSYNYGYWGQGTQVTVSSSGSMFDLTVRRNHLIEDVLNQLSQFENEDLRKELWVSFSGEIGYDLGGVKKEFFYCLFAEMIQPEYGMFMYPEGASCMWFPVKPKFEKKRYFFFGVLCGLSLFNCNVANLPFPLALFKKLLDQMPSLEDLKELSPDLGKNLQTLLDDEGDNFEEVFYIHFNVHWDRNDTNLIPNGSSITVNQTNKRDYVSKYINYIFNDSVKAVYEEFRRGFYKMCDEDIIKLFHPEELKDVIVGNTDYDWKTFEKNARYEPGYNSSHPTIVMFWKAFHKLTLEEKKKFLVFLTGTDRLQMKDLNNMKITFCCPESWNERDPIRALTCFSVLFLPKYSTMETVEEALQEAINNNRGFG

### Cell lines and culture

HEK293T human embryonic kidney cell line, A549 human lung adenocarcinoma epithelial cell line, HeLa human cervical cancer cell line, HCT116 colon cancer cell line, HT22 mouse hippocampal neuronal cell line, and BV2 mouse microglial cell lines were obtained from American Type Culture Collection and China Center for Type Culture Collection. HEK293T, HeLa, A549, HCT116, and HT22 cells were cultured in Dulbecco's modified Eagle's medium supplemented with 10% fetal bovine serum (FBS) (hyclone), 100  U/ml penicillin, and 100 μg/ml streptomycin. BV2 cells were maintained in MEM with 10% FBS (hyclone), 100 U/ml penicillin, 100 μg/ml streptomycin. All cell lines were cultivated at 37 °C in 5% CO_2_ humidity. Early passage cell lines (<passage 25) were used. Plasmids or siRNA were transfected into cells according to Thermo Fisher protocol (L3000015, Lipofectamine 3000 Reagent). The siRNA sequences used in the study are listed in Table S3.

### CRISPR/Cas9 gene knockout of eIF4E2 in HT22 cells

sgRNAs-targeting eIF4E2 gene were cloned into pX330-U6-Chimeric_BB-CBh-hSpCas9 (Addgene, #42230) as previously described (5). sgRNA sequences are as follows: CAGCCTGCCTGGCATTCTAGtgg. HT22 cells were cotransfected with plasmid pX330 and cas9 Nuclease (GenScript, Z03386-50) containing sgRNAs-targeting eIF4E2. Single-cell clones were selected with puromycin and tested for positive by using PCR, using primers P1: GAGGTCTGCCTCTGGGACTT/P2: CGATGACGCCAGCTTCAAT and two individual clones were used for further experiments.

### GST pull-down assay

GST pull-down assay was performed as previously described (46). The recombinant His or GST-tagged proteins were expressed in bacteria BL21 and purified by using Ni-NTA (Qiagen) and glutathione sepharose beads (GenScript), respectively. For GST pull-down assay, 1 nM of recombinant His_6_-tagged proteins and 1 nM of recombinant GST-tagged proteins were incubated in GST pull-down buffer (50 mM Hepes pH 7.5, 50 mM NaCl, 2 mM EDTA pH 8.0, 0.1% Nonidet P-40 [NP-40], and 10% glycerol) for 2 h at 4 °C, followed by precipitation with glutathione-sepharose beads for 30 min at 4 °C. After three rinses, beads were resuspended in SDS-PAGE sample buffer and subjected to IB analysis.

### Ni-NTA-agarose purification

Ni-NTA-agarose purification were performed as described previously (15) with some modifications. In brief, 36 h posttransfection, cells were washed with PBS and lysed with lysis buffer B (PBS containing 1% NP-40, 20 mM imidazole and protease inhibitor cocktail) for Ni-NTA-agarose purification. Ni-NTA agarose beads (20 ml) (Qiagen) were then added to cell lysates and rotated for 1 h at 4 °C. After three rinses with PBS containing 1% NP-40 and 20 mM imidazole, Precipitates were boiled in SDS-PAGE sample buffer and then subjected to IB analysis.

### *In vitro* ISGylation and kinase assay

*In vitro* ISGylation and kinase assay were performed as described previously (25, 26) with some modifications. To assess the impact of eIF4E2 ISGylation on the kinase activity of GSK3β (S/T-P), we employed various methods to purify the relevant protein components. The TNT rapid coupled transcription/translation system (Promega) was used to express ISGylation components (UBE1L, UBCH8, and HERC5 proteins). Cell lysates were incubated with anti-FLAG antibody agarose beads for 4 h to pull down FLAG-tagged proteins (FLAG-eIF4E2 (WT/4KR), FLAG-GSK3β). First, we incubated eIF4E2, HERC5, UBCH8, UBE1L, and ISG15 in an ATP-containing buffer at 37 °C for 1 h to achieve eIF4E2 ISGylation. Following this reaction, we combined the resulting components with GSK3β and p53 in an ATP-containing buffer and incubated them at 30 °C for 1 h to perform an *in vitro* kinase assay. Equal amounts of lysates were separated by SDS-PAGE and transferred onto nitrocellulose membranes for IB analysis.

### MCAO/R model establishment

Focal ischemia was performed as described previously (49) with some modifications. Select male C57BL/6 mice aged 7 to 8 weeks and weighing 22 to 24 g to construct a mouse MCAO/R model, with the control group undergoing sham surgery. The specific steps are as follows: mice were anesthetized with a mixture of isoflurane, and the midline of the neck was incised to expose the right common carotid artery branch in a mixture of 30% oxygen and 70% nitrous oxide. Then, the right external carotid artery was ligated, and a smooth, round-tipped nylon suture was inserted into the right internal carotid artery through the bifurcation of the right external carotid artery and common carotid artery, blocking the right middle cerebral artery at a distance of 10 ± 0.5 mm. Reperfusion (24 h) was achieved by removing the suture after 1 h. The control group mice underwent a similar sham surgery, with the difference being that the suture was inserted at a distance less than 8 mm to avoid occlusion. During the surgery, a heating pad was used to keep the animals warm, maintaining body temperature at 36.5 to 37.5 °C. Meloxicam was administered for postoperative analgesia. The success of the MCAO/R model establishment was identified by immediate detection of scattered tissue blood flow using transcranial laser Doppler, *ex vivo* infarct volume detection using magnetic resonance imaging, and 2,3,5-triphenyltetrazolium chloride staining.

### OGD/R model establishment

Initiate the cell culture by seeding the cells into a 12-well plate, allocating three wells per experimental group. Adjust the seeding density to 100,000 cells per well, targeting an initial 10% confluence, and incubate for 24 h to achieve approximately 60% confluence. Subsequently, remove the spent medium, rinse the wells with PBS once, and supplement each well with 400 μl of a customized medium: sugar-free, devoid of phenol red, and fortified with 10% FBS and 1% antibiotics. Subject the OGD group to hypoxic conditions within an incubator for a 2-h treatment period. Post OGD treatment, aspirate the sugar-free medium and replace it with a standard medium, also free of phenol red, containing 10% FBS and 1% antibiotics, for an additional 6, 12 or 24-h incubation. After this interval, discard the medium, rinse with PBS, and introduce 500 μl of a 20 μM 2′,7′-dichlorofluorescin diacetate solution to each well. Allow the cells to incubate for 30 min. Following this, perform two PBS rinses and apply 500 μl of a 4% formaldehyde fixative to each well, fixing the cells at room temperature for 15 min. Next, conduct a single PBS rinse and add 500 μl of DAPI staining solution to each well, incubating at room temperature for another 15 min. After two final PBS rinses, apply mounting medium to the slides, ensure they are dried in a dark environment, and prepare the slides for subsequent imaging procedures.

### RNA isolation and RT-PCR

Total RNA was extracted using TRIzol (Invitrogen) according to manufacturer’s instructions. M-MuLV reverse transcription (Takara) was used for mRNA measurements. In brief, reverse transcription was performed using the ExScript reverse transcription reagent kit (Takara) in a final volume of 20 ml containing 1 μg total RNA, 4 ml 5X ExScript buffer, 1 ml deoxynucleotide triphosphate (10 μM) mixture, 1 ml oligo(dT) primer, 0.5 ml ExScript RTase, 0.5 ml RNase inhibitor, and RNase-free water. PCR was conducted according to the instructions of TsingKe Golden mix under the following conditions: pre-DNA denaturation at 98 °C for 2 min; DNA denaturation at 98 °C for 10 s; annealing for 30 s at 60 °C; elongation was carried out at 72 °C for 10 s; the total cycle number was 30. All experiments were performed in triplicate. Normalization was made to total RNA and *GAPDH* mRNA with similar results. The data presented are the ones normalized to *GAPDH* transcripts. The qPCR primers are listed in Table S4.

### Statistical analyses

Statistical analyses and data visualization were performed using GraphPad Prism 9 (GraphPad Software; https://www.graphpad.com/). All quantitative experiments were done in triplicate unless otherwise indicated. Data are presented as the mean ± SD. The significance was expressed with ∗: *p* < 0.05, ∗∗: *p* < 0.01, and ∗∗∗: *p* < 0.001.

### Ethics

All animal experiments were approved by the Institutional Animal Care and Use Committee of Huazhong Agricultural University (approval number: HZAUMO-2024-0311).

## Data availability

Data will be made available on request.

## Supporting information

This article contains supporting information.

## Conflict of interest

The authors declare that they have no conflicts of interest with the contents of this article.

## Figures and Tables

**Supporting Figure S4:**