# Supplementary material for: NITAC-mediated ISGylation of eIF4E2 attenuates GSK3β proline-directed kinase activity, conferring cytoprotection
Source: J Biol Chem. 2025 Sep 27;301(11):110777. doi: 10.1016/j.jbc.2025.110777 (PMC12607020; doi:10.1016/j.jbc.2025.110777)
Supplement: Supporting Information [file mmc1.docx]

**Supporting Information For**

NITAC-Mediated ISGylation of eIF4E2 Attenuates GSK3β Proline-Directed Kinase Activity, Conferring Cytoprotection

**Supplementary Tables**

**Table S1 Reagent or Resource**

| **REAGENT** | **SOURCE** | **IDENTIFIER** |  |
| --- | --- | --- | --- |
| **Antibodies** | | |  |
| Actin | Sigma Aldrich | F3022 |  |
| FLAG-Tag | Sigma Aldrich | F4799 |  |
| HA-Tag | ABclonal | AE008 |  |
| GST-Tag | ABclonal | AE001 |  |
| His-Tag | ABclonal | AE003 |  |
| Myc-Tag | Proteintech | 60003-2-Ig |  |
| HRP Goat Anti-Rabbit IgG | ABclonal | AS014 |  |
| HRP Goat Anti-Mouse IgG | ABclonal | AS003 |  |
| GSK3β | Santa Cruz Biotechnology | sc-81462 |  |
| HERC5 | ABclonal | A14889 |  |
| UBE1L | ABclonal | A9142 |  |
| ISG15 | Proteintech | 15981-1-AP |  |
| UBCH8 | ABclonal | A13670 |  |
| Hif1α | Abcam | ab308433 |  |
| p53 | Abcam | ab26 |  |
| p-p53 (S315) | Cell Signaling Technology | #2528 |  |
| p-p53 (S33) | ABclonal | AP0762 |  |
| p-p53 (S46) | ABclonal | AP0476 |  |
| p-p53 (S376) | Abcam | ab183547 |  |
| Bcl2 | Abcam | ab32124 |  |
| Bax | Abcam | ab32503 |  |
| Cleaved-Caspase3 | Abcam | ab2302 |  |
| Goat Anti-Rabbit IgG H&L (FITC) | Abcam | ab6717 |  |
| Goat Anti-Mouse IgG H&L (Alexa Fluor® 647) | Abcam | ab150115 |  |
| **Kit** |  |  |  |
| Bradford Protein Assay | Beyotime | P0006C |  |
| CCK8 | Beyotime | C0038 | |
| **Chemicals** |  |  |  |
| DCFH-DA | Sigma | 2044-85-1 |  |
| IFN-β Protein, Human (CHO) | MCE | HY-P73128 | |

**Table S2 PCR-Primers for Genes**

| **Gene ID No** | **Forward primer (5'–3')** | **Reverse primer (5'–3')** |
| --- | --- | --- |
| *ISG15* | AAAAGGATCCATGGGCTGGGACCT | AAAACTCGAGTTAGCTCCGCCCG CCAGG |
| *His_6_-ISG15* | AAAAGGATCCATGGGCTGGGACCT | AAAACTCGAGTTAGCTCCGCCCG CCAGG |
| *UBE1L* | AAAAAAGCTTATGGATGCCCTGGACGCTTC | AAAACTCGAGTCACAGCTCATAGTGCAGAGGTG |
| *UBCH8* | AAAAGGATCCATGATGGCGAGCATGCGAGTG | AAAACTCGAGTTAGGAGGGCCGGTCCACTCC |
| *HA-HERC5* | AAAAGGATCCATGGAGCGGAGGTCGCGG | AAAACTCGAGTCAGCCAAATCCTCTGTTGTTGTTG |
| *SPOP* | GACTACCGGTAGCGTGAACATCTCCGGCCAG | TTCTGCGGCCGCTCAGCTCTGTTTCAGTCTCTTCCTGG |
| *FLAG-Nb.BV025-SPOP* | AAAAGGATCCCAGGTGCAGCTGCAGGAGTCT | AAAAACCGGTTGAGGAGACGGTGACCTG |
| *FLAG-Nb.28E11-SPOP* | AAAAGGATCCCAGGTGCAGCTGCAGGAGTCT | AAAAACCGGTTGAGGAGACGGTGACCTG |
| *FLAG-Nb.30C7-SPOP* | AAAAGGATCCCAGGTGCAGCTGCAGGAGTCT | AAAAACCGGTTGAGGAGACGGTGACCTG |
| *His_6_-Nb.BV025-SPOP* | GACTGAATTCATGCAGGTGCAGCTGCAGGAGTC | GACTCTCGAGCTATGAGGAGACGGTGACCTGAG |
| *His_6_-Nb.28E11-SPOP* | GACTGAATTCATGCAGGTGCAGCTGCAGGAGTC | GACTCTCGAGCTATGAGGAGACGGTGACCTGAG |
| *His_6_-Nb.30C7-SPOP* | GACTGAATTCATGCAGGTGCAGCTGCAGGAGTC | GACTCTCGAGCTATGAGGAGACGGTGACCTGAG |
| *GST-eIF4E2* | GACTGGATCCATGAACAACAAGTTCGACGC | GACTCTCGAGTCATGGCACATTTTGGGGGCCCAGCCTGCCT |
| *HOTag3-eIF4E2* | GACTGGATCCATGAACAACAAGTTCGACGC | GACTGAATTCTGGCACATTCAACCGCG |
| *HOTag6-GSK3β* | GACTGGTACCATGTCAGGGCGGCCCAGAA | GACTACCGGTGGTGGAGTTGG AAGCTGATGCAG |
| *HA-RBR* | ATTGGATCCATGCCTTGTCAGATCTGCT | ATTCTCGAGATTATAGCGGTTACAGTTGTACC |
| *HA-HECT* | ATTGGATCCATGCCTTGTCAGATCTGCT | ATTCTCGAGTCAGCCAAATCCTCTGTT |
| *HA-Nb.28E11-RBR* | ATTAAGCTTCAGGTGCAGCTGCAGGA | ATTCTCGAGATTATAGCGGTTACAGTTGTACC |
| *HA-Nb.30C7-RBR* | ATTAAGCTTCAGGTGCAGCTGCAGGA | ATTCTCGAGATTATAGCGGTTACAGTTGTACC |
| *HA-Nb.28E11-HECT* | ATTAAGCTTCAGGTGCAGCTGCAGGA | ATTCTCGAGTCAGCCAAATCCTCTGTT |
| *HA-Nb.30C7-HECT* | ATTAAGCTTCAGGTGCAGCTGCAGGA | ATTCTCGAGTCAGCCAAATCCTCTGTT |

**Table S3 siRNA-Primers for Genes**

| **Gene ID No** | **SenseSeq (5'–3')** | | |
| --- | --- | --- | --- |
| *eIF4E2* | #1 (5′CUCACACCGACAGCAUCAATT3′) | #2 (5′CACAGAGCUAUGAACAGAAUATT3′) | |
| *ISG15* | #1 (5′GCAACGAAUUCCAGGUGUCTT3′) | | #2 (5′GAGCACCGUGUUCAUGAAUTT3′) |

**Table S4 QPCR-Primers for Genes**

| **Gene ID No** | **Forward primer (5'–3')** | **Reverse primer (5'–3')** |
| --- | --- | --- |
| *IL-1* | GCAACTGTTCCTGAACTCAACT | ATCTTTTGGGGTCCGTCAACT |
| *IL-6* | TAGTCCTTCCTACCCCAATTTCC | TTGGTCCTTAGCCACTCCTTC |
| *TNF-α* | TCTCAGCCTCTTCTCATTCCTGCT | AGAACTGATGAGAGGGAGGCCATT |
| *TGF-β* | GGTCTCAACCCCCAGCTAGT | GCCGATGATCTCTCTCAAGTGAT |
| *IL-10* | GCTCTTACTGACTGGCATGAG | CGCAGCTCTAGGAGCATGTG |
| *IL-4* | TCTGCATTGCACTTATGCTGA | AAAGGGCGATCTAGTGATGGA |
| *GAPDH* | AGGTCGGTGTGAACGGATTTG | TGTAGACCATGTAGTTGAGGTCA |

**Figure Legends for Supplementary Figures**

**Supplemental Figure 1.** **NITAC-Enabled Activation of eIF4E2 ISGylation**

(S1A) (S1B) The HECT and RBR domains directly promote eIF4E2 ISGylation. HEK293T cells were transfected with plasmids expressing the His_6_-ISGylation system. Cell lysates were subjected to Ni-NTA pull-down and IB analysis.
(S1C) NITAC-targeted activation of exogenous FLAG-eIF4E2 ISGylation. HEK293T cells were transfected with plasmids expressing FLAG-eIF4E2, HA-HECT or NITAC (HA-Nb-HECT) that incorporates eIF4E2-nanobodies linked to HECT, and the His_6_-ISGylation system as indicated. Cell lysates were subjected to Ni-NTA pull-down and IB analysis.
(S1D) NITAC-targeted activation of endogenous eIF4E2 ISGylation. HEK293T cells were transfected with plasmids expressing HA-HECT or NITAC (HA-Nb-HECT) and the His_6_-ISGylation system as indicated. Cell lysates were subjected to Ni-NTA pull-down and immunoblotting analysis.

**Supplemental Figure 2. ISGylation of eIF4E2 Enhances Its Interaction with GSK3β**

(S2A) A schematic representation of the eIF4E2 structure, showing its ISGylation sites and the positioning of the GSK3β binding domain.

(S2B) A schematic diagram illustrating the SPPIER principle.

(S2C) (S2D) ISGylation promotes the interaction between eIF4E2 and GSK3β. (S2C) HeLa cells were transiently transfected with plasmids expressing eIF4E2-EGFP-HOTag3-T2A-HOTag6-GSK3β or eIF4E2(4KR)-EGFP-HOTag3-T2A-HOTag6-GSK3β fusion proteins and treated with IFNβ (1000 U/mL) for 48 hours. Fluorescence microscopy images were captured. Scale bar = 2 μm. (S2D) HEK293T cells were transfected with plasmids expressing the ISGylation system (UBE1L, UBCH8, and ISG15), HHARI and eIF4E2 (WT/4KR)-EGFP-HOTag3-T2A-HOTag6-GSK3β for 48 hours. Fluorescence microscopy images were captured. Scale bar = 2 μm.

(S2E) The lysine-to-arginine (KR) mutants in eIF4E2 do not affect its binding to GSK3β. HEK293T cells were transfected with plasmids expressing FLAG-tagged GSK3β and HA-tagged eIF4E2 (including wild-type and KR mutants). After 48 hours, cell lysates were subjected to immunoprecipitation with an anti-Flag antibody, followed by IB analysis.

(S2F) eIF4E2-ISG15 enhances the binding of eIF4E2 to GSK3β. HEK293T cells were transfected with plasmids expressing Flag-ISG15, Flag-eIF4E2 and Flag-eIF4E2-ISG15. After 48 hours, cell lysates were subjected to immunoprecipitation with an anti-Flag antibody, followed by IB analysis.

(S2G) eIF4E2-ISG15 cannot regulate the proline-directed kinase activity of GSK3β. HEK293T cells were transfected with plasmids expressing Flag-eIF4E2 and Flag-eIF4E2-ISG15. After 48 hours, cell lysates were subjected to immunoprecipitation with an anti-Flag antibody, followed by IB analysis.

**Supplemental Figure 3. NITAC-Enabled eIF4E2 ISGylation Provides Cytoprotection post OGD/R**

(S3A) Nb.RBM47-NITAC cannot active ISGylation of endogenous eIF4E2. HT22 cells were transfected with plasmids expressing HA-HECT, Nb.RBM47-NITAC or Nb.30C7-NITAC and subjected to 2 hours of OGD followed by 6 hours of reoxygenation. Cell lysates were analyzed by IB.

(S3B) Nb.RBM47-NITAC cannot active ISGylation of endogenous eIF4E2. HT22 cells were transfected with plasmids expressing HA-HECT, Nb.RBM47-NITAC or Nb.30C7-NITAC and subjected to 2 hours of OGD followed by 6 hours of reoxygenation. ROS levels were measured using DCFH-DA staining, followed by fluorescence microscopy. Scale bar = 30 μm. Data are presented as the mean ± SD. Statistical significance was determined by one-way ANOVA (n = 3 for each group, *: *p<* 0.05, **: *p<* 0.01, ***: *p<* 0.001).

(S3C) Knockout of *eIF4E2* inhibits the S/T-P phosphorylation of downstream substrates of the eIF4E2-GSK3β pathway. Lysis of *eIF4E2*-KO HT22 (KO) and isogenic wild-type HT22 (WT) cells, followed by IB analysis.

(S3D) (S3E) Nb.30C7-NITAC did not cause changes in protein levels related to apoptosis (S3D) and cell survival (S3E) in *eIF4E2*-KO HT22 cell. Data were expressed as mean ± SD. Statistical significance was determined by one-way mean ANOVA (n = 3 per group, *: *p<* 0.05, **: *p<* 0.01, ***: *p<* 0.001).

(S3F) Nb.30C7-NITAC did not cause changes in ROS levels in *eIF4E2*-KO HT22 cell. ROS levels were measured using DCFH-DA staining, followed by fluorescence microscopy. Scale bar = 30 μm. Data are presented as the mean ± SD. Statistical significance was determined by two-way ANOVA (n = 3 for each group, *: *p<* 0.05, **: *p<* 0.01, ***: *p<* 0.001) (Shown in the right panel).

**Supplemental Figure 4. Densitometric analysis of western blot results presented in Figure 3 and Figure S1**

(S4A) (S4B) (S4C) (S4D) (S4E) (S4F) Densitometric analysis of western blot results. Densitometric analysis of western blot results presented in Figure 3B (S4A), Figure 3C (S4B), Figure 3E (S4C), Figure 3F (S4D), Figure S1C (S4E), S1D (S4F). Data were expressed as mean ± SD. Statistical significance was determined by one-way mean ANOVA (n = 3 per group, *: *p<* 0.05, **: *p<* 0.01, ***: *p<* 0.001).
